# Generation of Novel High-Quality Small-Grained Rice Germplasm by Targeting the *OsVIN2* Gene

**DOI:** 10.3390/biology15010064

**Published:** 2025-12-30

**Authors:** Xi Chen, Yarong Lin, Xiangzhe Xi, Shaohua Yang, Shiyu Wu, Hongge Qian, Mingji Wu, Taijiao Hu, Fating Mei, Mengyan Zheng, Chuanlin Shi, Yiwang Zhu

**Affiliations:** 1 Fujian Provincial Key Laboratory of Agricultural Genetic Engineering, Biotechnology Research Institute, Fujian Academy of Agricultural Sciences, Fuzhou 350003, China; chenxi02@caas.cn (X.C.); yarong_lin@126.com (Y.L.); 13587513880@163.com (X.X.); ysh@fjage.org (S.Y.); qianhg0706@stu.hunau.edu.cn (H.Q.); wmj@fjage.org (M.W.); htj@fjage.org (T.H.); 13516377413@163.com (F.M.); 2 Key Laboratory of Crop Physiology, Ecology and Genetic Breeding, Ministry of Education, Jiangxi Agricultural University, Nanchang 330045, China; 3 Shenzhen Branch, Guangdong Laboratory of Lingnan Modern Agriculture, Genome Analysis Laboratory of the Ministry of Agriculture and Rural Affairs, Agricultural Genomics Institute at Shenzhen, Chinese Academy of Agricultural Sciences, Shenzhen 518124, China; wsy0908tt@163.com (S.W.); 15057532811@163.com (M.Z.)

**Keywords:** CRISPR/Cas9, germplasm improvement, grain quality, *OsVIN2*, small grain size

## Abstract

Small-grained rice varieties are highly valued in hybrid seed production and food markets because of their unique advantages in mechanized seed production and cooking qualities. Developing new varieties combining small grain size with high nutritional and cooking quality represents an important breeding objective. The *OsVIN2* gene has been identified as a key regulator involved in carbohydrate metabolism and grain development in rice. In this study, a CRISPR/Cas9-mediated gene-editing approach was employed to precisely modify the *OsVIN2* gene in the restorer line MH86 of high-quality rice varieties. Compared with the wild-type plants, the frameshift mutant seeds showed a significant reduction in length and width by 19.9% and 15.2%, respectively, leading to a 39.2% decrease in thousand-grain weight. Notably, the mutant exhibited improved quality traits, including a decrease of 16.6% in amylose content, an increase of 11.0% protein, and a 77.5% and 84.7% decrease in chalkiness rate and chalkiness degree, respectively. These results demonstrate that targeted editing of *OsVIN2* is a promising approach for creating novel small-grained rice germplasm with superior quality attributes.

## Simple Summary

Small-grained rice is valuable for hybrid seed production and food markets, but developing high-quality small-grained varieties is challenging. This study aimed to use the CRISPR/Cas9 gene-editing technique to modify the *OsVIN2* gene in rice variety MH86, hoping to reduce grain size while improving quality. We successfully edited *OsVIN2*, resulting in rice grains that were shorter and narrower than normal. The edited rice also had better quality: less chalkiness, more protein (11.0%), and optimized cooking texture. Importantly, other plant traits like growth and seed-setting remained unaffected. This method provides an effective way to breed high-quality small-grained rice, which can lower hybrid seed production costs and meet consumer demand for better-tasting rice.

## 1. Introduction

Rice grain size, a key agronomic trait, not only determines grain yield but also influences grain quality and application scenarios [1,2]. Among diverse grain size variants, small-grained rice germplasm represents a crucial component of rice genetic diversity. It provides valuable genetic resources for dissecting the molecular regulatory network of grain shape and also has significant practical value in hybrid rice seed production [1,2,3]. Specifically, small-grained male sterile lines can reduce the male-to-female seed ratio during mechanical mixing, lower seed production costs, and improve seed propagation efficiency [4]. Additionally, small-grained rice meets diversified market demands, as it is preferred for traditional snacks and high-quality congee in some regions [5,6]. Thus, developing efficient strategies to create high-quality small-grained rice germplasm is needed in rice breeding.

In recent years, CRISPR/Cas9-mediated gene editing technology has revolutionized crop germplasm innovation due to its high efficiency, precision, and simplicity [7]. This technology has been widely applied to modify grain size-related traits in rice. For example, targeted mutation of *GS3* (a major negative regulator of grain length) or *GW2* (a negative regulator of grain width) via CRISPR/Cas9 increased grain length or width effectively in many rice varieties [8,9,10], confirming that CRISPR/Cas9 is a powerful tool for grain size modification.

To date, numerous genes regulating rice grain size have been identified, but their application in small-grained germplasm creation remains limited. This is mainly due to two constraints. On the one hand, a large proportion of the identified grain size-related genes are negative regulators [11]. Their loss-of-function mutations typically increase grain size, rendering them unsuitable for creating small-grained varieties via gene editing. On the other hand, the few positive regulators that can reduce grain size when edited often exert adverse effects on other agronomic traits. For instance, the mutation of the *OsCSLD4* gene reduced grain size, but it led to side effects on agronomic traits such as poor plant growth, reduced plant yield, and decreased bacterial blight resistance [12]. Therefore, the key challenge lies in selecting suitable target genes that can synchronously optimize grain size and quality without adverse effects on other agronomic traits.

*OsVIN2* could be an optimal candidate target gene for creating small-grained rice germplasm. Two independent studies have demonstrated that loss of *OsVIN2* function can effectively reduce grain size [13,14]. *OsVIN2* encodes a vacuolar invertase, which influences sugar composition, transport, and starch accumulation [13,14,15]. Specifically, in *OsVIN2* loss-of-function mutants, starch composition is altered significantly [13]. Since starch accumulation is a key determinant of rice eating quality and affects various traits such as amylose content (AC), gelatinization temperature (GT), gel consistency (GC), chalkiness rate (CR), and chalkiness degree (CD) [16,17,18], it is worth further investigating whether editing *OsVIN2* impacts rice eating quality.

MH86 is a widely used indica restorer line in hybrid rice production, with high combining ability, strong disease resistance, and good grain quality [19,20]. However, its grain size is larger than the ideal small-grained type for mechanical seed production. Notably, gene function in rice often shows genetic background dependence. For example, knockout of *QSOXL1* significantly reduces seed dormancy levels in japonica varieties, Nanjing7, and Nanjing35, but has no effect on seed dormancy in N22 (an Aus rice variety) [21]. Whether *OsVIN2* editing can modify grain size and quality in MH86 remains untested, and thus experimental validation is needed to clarify its efficacy in this genetic background.

In this study, we aim to edit the rice gene *OsVIN2* using CRISPR/Cas9 technology to develop new quality-improved germplasm resources of small-grain rice. We hypothesize that precision modification of *OsVIN2* can simultaneously reduce grain size and improve quality traits, such as amylose content. This research provides a new approach for breeding small-grain varieties, holding important implications for broader crop improvement strategies.

## 2. Materials and Methods

### 2.1. Plant Material and Growth Conditions

The elite indica rice variety Minghui86 (MH86) was used as the wild-type (WT) control and transformation recipient. All WT and transgenic plants were grown in the experimental field of Fuzhou Agricultural Station (26.08° N, 119.28° E), Fujian Province, under natural conditions. The growing season spanned from late April to early October, with standard agronomic management including regular irrigation, fertilization, and pest control. For trait stabilization, 15 T_2_ homozygous mutants were selected for phenotypic analysis to avoid segregating effects.

### 2.2. sgRNA Design and Vector Construction

The *OsVIN2* gene (*Os02g0106100*) sequence of MH86 was retrieved from the Rice Genome Annotation Project (RGAP, http://rice.plantbiology.msu.edu/, accessed on 28 December 2025). A 20-bp sgRNA target sequence (5′-TGGCGGTCCTCTCCGGCGTC-3′) was designed in the first exon using CRISPR-P 2.0 (http://skl.scau.edu.cn/targetdesign/, accessed on 28 December 2025) [22], ensuring high specificity and minimal off-target potential. Complementary oligonucleotides (Table S1) were synthesized, annealed to form dimers, and cloned into the CRISPR/Cas9 vector pYLCRISPR/Cas9 [23], which contains the Cas9 gene driven by the maize Ubiquitin promoter and the sgRNA under the rice U6 promoter.

### 2.3. Agrobacterium-Mediated Rice Transformation

The recombinant vector was introduced into Agrobacterium tumefaciens strain EHA105 via electroporation. Calli induced from mature embryos of MH86 were infected with the positive Agrobacterium cultures. Transformation procedures, including callus induction, co-cultivation, selection (hygromycin 50 mg/L), and plant regeneration, followed the protocol described by Hiei et al. [24]. Regenerated T_0_ plants were transplanted to the field and selfed to produce T_1_ and T_2_ generations.

### 2.4. Genotyping and Off-Target Analysis

Genomic DNA was extracted from young leaves using the CTAB method [25]. The target region of *OsVIN2* was amplified with specific primers (F: 5′-CGCTTCCCTACTCCTACTCG-3′, R: 5′-CATCAGCCTCACCATCTCCT-3′) (Table S1) under the following conditions: 94 °C for 5 min; 35 cycles of 94 °C for 30 s, 58 °C for 30 s, 72 °C for 30 s; and 72 °C for 10 min. PCR products were Sanger-sequenced, and mutation types were identified by comparing with WT sequences using DNAMAN 9.0. For off-target detection, 6 potential sites predicted by CRISPR-P were amplified and sequenced in homozygous mutants.

### 2.5. Agronomic Trait Measurement

At maturity, 15 T_2_ plants from each mutant line (*osvin2*-#4, #19) and WT were randomly selected for trait analysis. Plant height (distance between the ground surface and the tip of the main panicle), tiller number(number of tillers at maturity), panicle length(distance between the rachis and the tip of the main panicle), and grain number per panicle were recorded (15 plants). Grain length and width were measured using a digital caliper (*n* = 300 grains per line). 1000-grain weight was determined using three replicates of fully filled grains. All data are presented as mean ± standard deviation (SD), and statistical significance was determined using Student’s *t*-test (*p* < 0.01).

## 3. Results

### 3.1. CRISPR/Cas9 Target Site Selection for the OsVIN2 Gene

**Figure 1:**
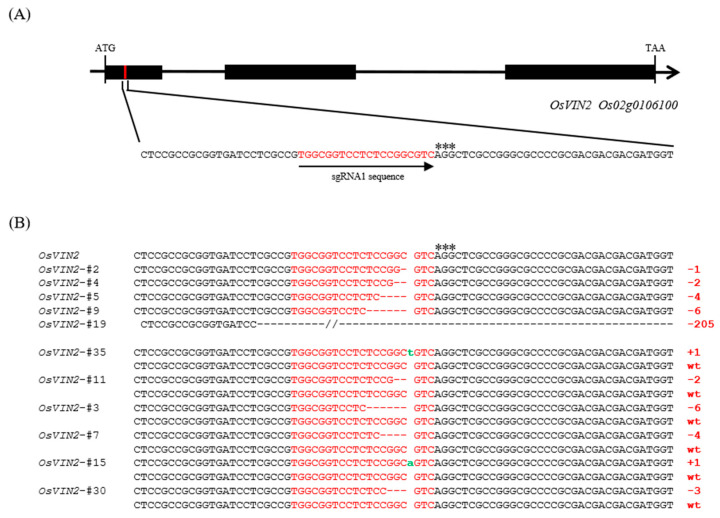
sgRNA Targeting Site and Genomic Sequence Variations in *osvin2* Mutants Relative to MH86. (**A**). The exons and sgRNA1 in the *OsVIN2* gene (*Os02g0106100*) sequence are indicated by black boxes and red font respectively, and protospacer adjacent motif (PAM) in gene is indicated asterisks. (**B**). The sequencing results of the T_0_ mutant of *OsVIN2* show that the inserted part is indicated by green letters, the number of missing bases is indicated by “-”, and the number on the right indicates the number of bases that have been reduced or inserted compared to *OsVIN2*. Those without insertion or reduction are wild type (WT).

Firstly, sequence analysis revealed that the *OsVIN2* gene contains three exons. Considering that the first exon of *OsVIN2* encodes a conserved domain critical for vacuolar invertase activity, we envision designing a target site within this region. Based on analysis from the prediction website, a sgRNA with high specificity was selected (Figure 1A). To ensure the suitability of this sgRNA in our selected material (MH86, which is an elite indica rice variety with high yield potential and moderate grain quality), we then amplified the sequence flanking this sgRNA in MH86. Sequence alignment results indicated that the sequence at this site in MH86 is identical to that in reference genome Nipponbare (Figure S1). Therefore, this sgRNA was prioritized for subsequent vector construction.

### 3.2. CRISPR/Cas9-Mediated Mutagenesis of OsVIN2

The CRISPR/Cas9 expression vector harboring sgRNA was introduced into MH86 calli via *Agrobacterium tumefaciens*-mediated transformation, and a total of 30 independent T0 transgenic plants were regenerated. Genomic DNA extracted from young leaves was used to amplify and identify the mutations of *OsVIN2* gene. Sequencing results showed that 18 plants (60.0% efficiency) carried mutations in *OsVIN2*, including 5 homozygous mutants, 6 biallelic mutants, and 7 heterozygous mutants (Table 1). Most mutations were small deletions (1–20 bp), accounting for 74.2% of edited alleles, while one line exhibited a large-fragment editing with a 205-bp deletion (Figure 1B; Table S2). These findings demonstrated efficient editing of *OsVIN2* using this sgRNA via the CRISPR/Cas9 system.

### 3.3. Putative Off-Target Analysis

Off-target effects have long been a major concern in the CRISPR/Cas9 system. Thus, evaluating the off-target effects by detecting mutations in potential off-target sequences remains an essential step. Therefore, we used the online tool OffTarget (http://skl.scau.edu.cn/offtarget/, accessed on 28 December 2025) to predict potential off-target sites of sgRNA-*OsVIN2*. Subsequently, 5 homozygous mutants with distinct mutation (*osvin2*-#2, #4, #5, #19, #9) were selected to assess the predicted off-target sequences. PCR identification results showed that no mutations were detected in the transgene-free mutant lines (Table 2, Figure S2). These results indicated that the designed sgRNA exhibits high specificity in inducing mutations in the *OsVIN2* gene, thereby ensuring the reliability of subsequent phenotypic analyses.

### 3.4. Phenotypic Analysis of Grain Size in OsVIN2 Mutants

**Figure 2:**
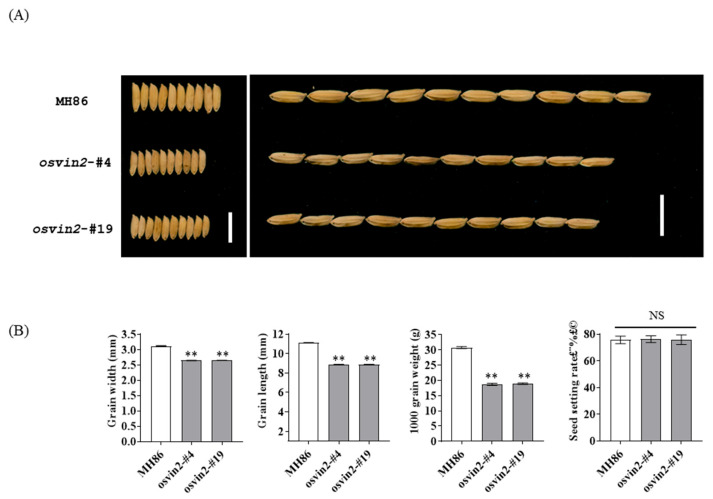
The grain phenotype differences between MH86 and *osvin2* mutants. (**A**). The morphology of rice grains after the growth of *osvin2* mutant plants. Scale bar, 1 cm. (**B**). Grain width, grain length, 1000-grain weight and seed setting rate of MH86 and osvin2 mutants. NS, no significant difference. Data are presented as mean ± SD. Student’s *t*-test was used to generate the *p*-values (** *p* < 0.01).

**Figure 3:**
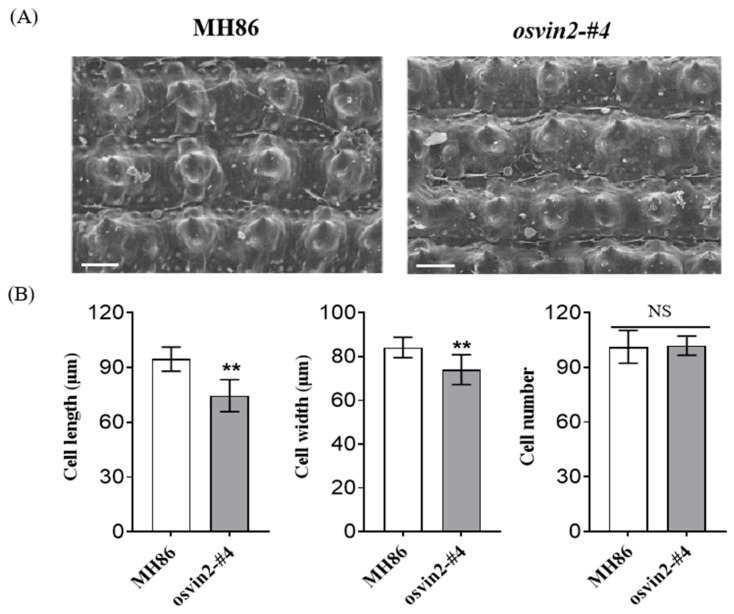
Scanning electron microscopic observation of spikelet lemma in MH86 and *osvin2* mutants. (**A**). Scanning electron microscopy was used to observe the outer epidermal cells of mature seeds of the *osvin2* mutant. Scale bar, 50 μm. (**B**). Presents the comparative analysis of outer integument cell characteristics among the above materials, specifically including, comparison of outer cell length, comparison of outer cell width, and comparison of outer cell number. NS, no significant difference. Data are presented as mean ± SD. Student’s *t*-test was used to generate the *p*-values (** *p* < 0.01).

To evaluate the effect of *OsVIN2* mutants on grain size, we measured grain dimensions of homozygous transgene-free T_2_ mutants (*osvin2*-#4, #19) and wild-type MH86. Compared with wild type, the mutant seeds showed a significant reduction in both length (19.9%) and width (15.2%), resulting in a 39.2% decrease in 1000-grain weight (Figure 2). Notably, the seed setting rate of *OsVIN2* mutants remained unaffected (Figure 2), indicating that the mutation specifically targeted grain size without influencing reproductive output. Scanning electron microscopy of the lemma and palea revealed that the outer epidermal cells of mutants were smaller than those of wild-type plants, with no significant change in cell number (Figure 3). For cell counting, 10 randomly selected regions per lemma were analyzed, and 20 grains per line were measured. This indicated that the reduced grain size in *osvin2* mutants was primarily caused by suppressed cell expansion rather than altered cell division—consistent with the role of vacuolar invertases in regulating cell turgor pressure [26]. These findings confirmed that *OsVIN2* effectively regulated cell expansion to determine grain size in rice.

### 3.5. osvin2 Mutants Confer Superior Grain Quality

**Figure 4:**
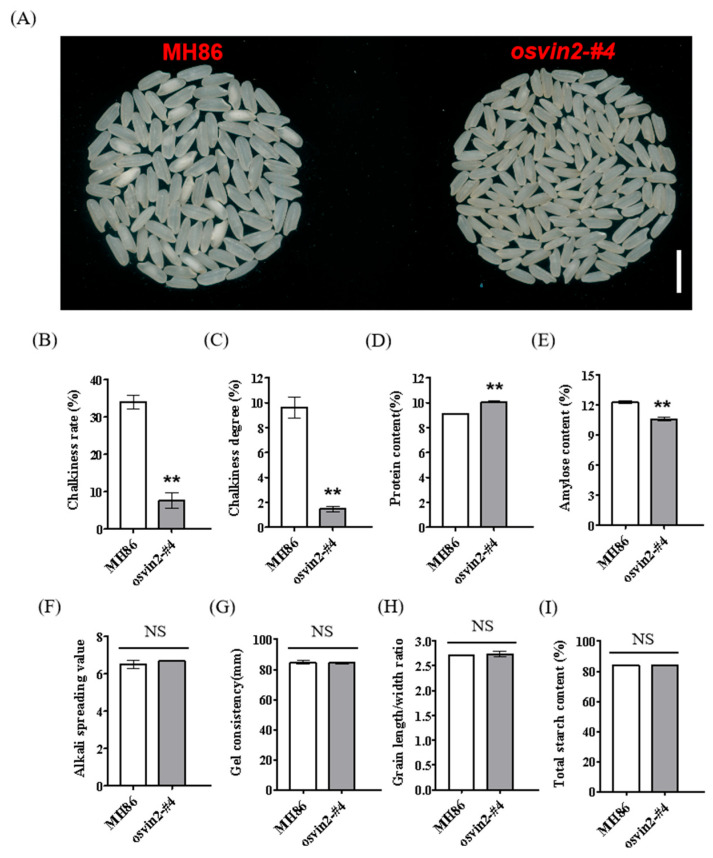
Comprehensive Comparison of Rice Grain Quality Traits between MH86 and *osvin2* mutants. (**A**) Appearance of polished grains of MH86 and *osvin2* mutants. Scale bar, 1 cm (**B**–**I**). The analysis and comparison charts of the chalkiness rate (**B**), chalkiness degree (**C**), protein content (**D**), amylase content (**E**), alkali spreading value (**F**), gel consistency (**G**), grain length/width ratio (**H**) and total starch content (**I**) of MH86 and osvin2 mutants are presented respectively. NS, no significant difference. Data are presented as mean ± SD. Student’s *t*-test was used to generate the *p*-values (** *p* < 0.01).

With growing consumer demand, rice quality has become a primary focus in rice breeding. This complex trait encompasses multiple dimensions including grain appearance, cooking properties, eating quality, and nutritional value. Accordingly, we analyzed key quality traits of polished grains from *osvin2* T_2_ mutants to evaluate improvements in grain quality. The results showed that compared with the wild type, *osvin2* mutant grains exhibited superior grain appearance, characterized by significantly reduced chalkiness rate and chalkiness degree (Figure 4A–C). Furthermore, the protein content of *osvin2* mutants was 10.1%, higher than the 9.1% in the wild type (Figure 4D). Amylose content (AC) is a critical factor determining rice’s cooking and eating quality. In general, high AC results in distinct, firmer grains, while lower AC yields a softer, stickier texture. Expectedly, the amylose content in *osvin2* mutants was noticeably lower than that in wild type (Figure 4E). However, no significant differences were observed in alkali spreading value, gel consistency, grain length/width ratio, or total starch content between *osvin2* mutants and the wild type (Figure 4F–I). Collectively, these analyses demonstrate that *osvin2* mutants exhibit improved rice quality, specifically manifested in enhanced visual appearance, increased protein content, and optimized amylose content.

### 3.6. Agronomic Traits of osvin2 Mutants

**Figure 5:**
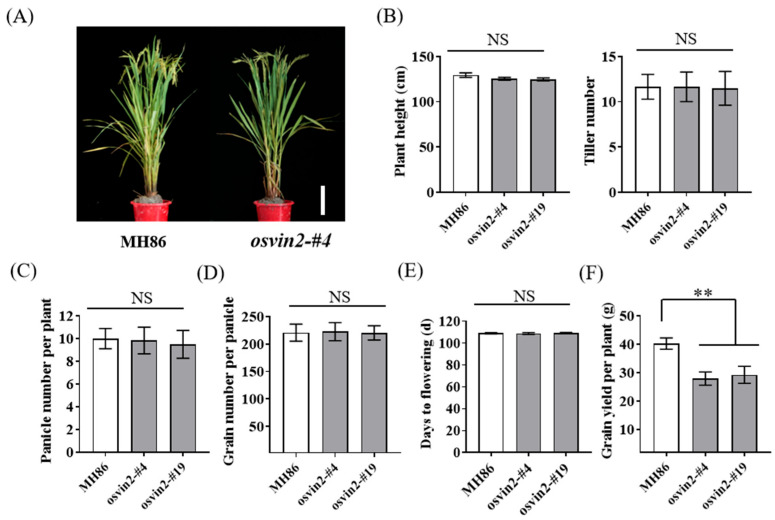
Plant Phenotypic Comparison and Analysis of Major Agronomic Traits between MH86 and *osvin2* mutants. (**A**) The plant phenotypes of MH86 and *osvin2* mutant. Scale bar, 20 cm. (**B**) Plant height, Tiller number of MH86 and *osvin2* mutants. (**C**–**F**) Panicle number per plant (**C**), Grain number per panicle (**D**), Days to flowering (**E**) and grain yield per plant (**F**) of MH86 and *osvin2* mutants. NS, no significant difference. Data are presented as mean ± SD. Student’s *t*-test was used to generate the *p*-values (** *p* < 0.01).

To assess the impact of *OsVIN2* on other agronomic traits, we investigated the field performance of T_2_ mutants. The results showed that the plant height and tiller number of *osvin2* mutants remained unchanged (Figure 5A,B). Additionally, panicle number per plant and grain number per panicle of *osvin2* mutant lines were comparable to those of the wild type (Figure 5C,D). Importantly, critical growth stages such as heading date and maturity were unaffected in *osvin2* mutants (Figure 5E), ensuring their adaptability to local cropping systems. Nevertheless, due to the reduced grain size and decreased 1000-grain weight of the *osvin2* mutants, this results in a significant reduction (27.3–30.3%) in grain yield per plant (Figure 5F). These findings indicated that *osvin2* mutants had minimal impact on overall plant architecture. The observed yield adjustment was a specific consequence of the targeted grain size modification, rather than a broad disruption of plant growth.

## 4. Discussion

The development of small-grained rice varieties holds significant practical value for advancing mechanized hybrid rice seed production. Their reduced grain size facilitates precise mechanical sorting during seed production, which can simplify large-scale cultivation, standardize seed quality, and reduce labor costs associated with traditional manual sorting [3,4]. Although this study did not directly measure mechanization efficiency, the size difference between mutant and WT grains (19.9% shorter and 15.2% narrower) provides a theoretical basis for mechanical separation. This study demonstrates that targeted editing of *OsVIN2* can simultaneously improve grain quality, including decreased amylose content and palatability, highlighting the multifaceted advantages of this approach.

Previous research cloned a completely recessive grain size QTL gene, *TGW5^FH212^*, which is a weak allele of the known *D1* gene. The introduction of TGW5^FH212^ converts conventional male sterile lines into small-grained sterile lines, increasing grain number while reducing grain size, thus providing a solution for low-cost, high efficiency mechanized hybrid rice seed production. However, it also results in a dwarf phenotype in *NIL^FH212^* [4]. In contrast, the *osvin2* mutants in this study only showed a significant improvement in grain shape (Figure 2 and Figure 3), without affecting other key agronomic traits such as tiller number, seed-setting rate, and flowering time (Figure 5). This minimal impact on overall plant architecture offers a feasible strategy for enhancing the efficiency of hybrid rice seed production.

The identification of *OsVIN2* as a positive regulator of grain weight provides a new molecular target for grain size regulation. Frameshift mutations in the first exon stably produce a small-grain phenotype, demonstrating that disrupting this vacuolar invertase gene is a feasible and actionable methodological approach for reducing grain weight without major pleiotropic effects. Unlike traditional breeding methods that often introduce linked undesirable traits, our approach enables targeted modification of grain size while improving rice quality without significantly compromising important agronomic characteristics such as plant type. The *OsVIN2*-edited lines developed in this study can be integrated into elite male sterile backgrounds to breed small-grained sterile lines suitable for mechanized hybrid seed production.

## 5. Conclusions

In summary, this study successfully demonstrated that CRISPR/Cas9-mediated targeted editing of the *OsVIN2* gene in the elite indica restorer line MH86 is an effective strategy for creating novel small-grained rice germplasm with superior quality attributes. By precisely modifying the first exon of *OsVIN2*, we achieved a significant reduction in grain size (19.9% shorter and 15.2% narrower) without compromising key agronomic traits such as tiller number, seed-setting rate, heading date, and plant architecture. Furthermore, the edited lines exhibited substantial improvements in grain quality, including increased protein content, and optimized amylose content. The high specificity of the designed sgRNA, confirmed by the absence of off-target mutations, ensures the reliability and safety of the edited germplasm for future breeding applications. This research not only identifies *OsVIN2* as a robust molecular target for synchronously regulating rice grain size and quality but also provides a practical framework for accelerating the development of small-grained rice varieties suitable for mechanized hybrid seed production.

## Figures and Tables

**Table 1:** T_0_ plants transformed with Cas9/sgRNA constructs targeting the homologous sequences of *OsVIN2* genes.

Variety	No. of Transgenic Plants	No. of Plants with Mutations (%)	No. of Homozygous Mutants (%)	No. of Biallelic Mutants (%)	No. of Heterozygous Mutants (%)
MH86	30	18 (60)	5 (16.6)	6 (20.0)	7 (23.3)

**Table 2:** Evaluation of potential off-target sites.

Target	Name of Putative Off-Target Sites	Putative Off-Target Locus	Putative Off-Target Sequence	No. of Mismatch Bases	No. of Plants Examined	No. of Indel Mutations
sgRNA	OFF1	chr01: 24753948	TGGTCGTCTTCTCCGGCGTC GGG	3	5	0
	OFF2	chr02: 21042203	TGAAGGTGCTCTCCGGCGTC AGG	3	5	0
	OFF3	chr01: 22880578	CGGCGATCCACTCCGGCGCC AGG	4	5	0
	OFF4	chr03: 35220013	TGGCGGTGGTCGCCGACGTC AGG	4	5	0
	OFF5	chr09: 15493407	TGCTCGTCCTCTTCGGCGTC AGG	4	5	0
	OFF6	Chr01: 26653040	GGGAGGTCCTCGCCGGCGAC AGG	4	5	0

## Data Availability

The original contributions presented in this study are included in the article/Supplementary Materials. Further inquiries can be directed to the corresponding authors.

## Supplementary Materials

The following supporting information can be downloaded at: https://www.mdpi.com/article/10.3390/biology15010064/s1, Figure S1: Exon sequence of the *OsVIN2* gene in MH86 material; It is consistent with the *Os02g0106100* gene sequence, indicating that the *OsVIN2* gene expression cassette in the edited material is intact without genomic variations that significantly affect gene function. Figure S2. The genomic locations and sequence information of potential off-target sitesOFF-target sites 1 to 6. Table S1. List of primers used in this study. Table S2. Genotypes of T_0_ mutant plants in MH86.

## References

[1] Gong D., Zhang X., He F., Chen Y., Li R., Yao J., Zhang M., Zheng W., Yu G. (2023). Genetic Improvements in Rice Grain Quality: A Review of Elite Genes and Their Applications in Molecular Breeding. Agronomy.

[2] Ren D., Ding C., Qian Q. (2023). Molecular bases of rice grain size and quality for optimized productivity. Sci. Bull..

[3] Lei B., Shao J., Zhang F., Wang J., Xiao Y., Cheng Z., Tang W., Wan J. (2023). Genetic analysis and fine mapping of a grain size QTL in the small-grain sterile rice line Zhuo201S. J. Integr. Agric..

[4] Ying J., Qin Y., Zhang F., Duan L., Cheng P., Yin M., Wang Y., Tong X., Huang J., Li Z. (2024). A weak allele of *TGW5* enables greater seed propagation and efficient size-based seed sorting for hybrid rice production. Plant Commun..

[5] Zhou H., Xia D., He Y. (2019). Rice grain quality—Traditional traits for high quality rice and health-plus substances. Mol. Breed..

[6] Ibarrola-Rivas M.J., Nonhebel S. (2022). Regional food preferences influence environmental impacts of diets. Food Secur..

[7] Guo Y., Zhao G., Gao X., Zhang L., Zhang Y., Cai X., Yuan X., Guo X. (2023). CRISPR/Cas9 gene editing technology: A precise and efficient tool for crop quality improvement. Planta.

[8] Huang J., Gao L., Luo S., Liu K., Qing D., Pan Y., Dai G., Deng G., Zhu C. (2022). The genetic editing of *GS3* via CRISPR/Cas9 accelerates the breeding of three-line hybrid rice with superior yield and grain quality. Mol. Breed..

[9] Rasheed H., Fiaz S., Khan M.A., Mehmood S., Ullah F., Saeed S., Khan S.U., Yaseen T., Hussain R.M., Qayyum A. (2022). Characterization of functional genes *GS3* and *GW2* and their effect on the grain size of various landraces of rice (*Oryza sativa* L.). Mol. Biol. Rep..

[10] Usman B., Zhao N., Nawaz G., Qin B., Liu F., Liu Y., Li R. (2021). CRISPR/Cas9 Guided Mutagenesis of Grain Size 3 Confers Increased Rice (*Oryza sativa* L.) Grain Length by Regulating Cysteine Proteinase Inhibitor and Ubiquitin-Related Proteins. Int. J. Mol. Sci..

[11] Chen K., Łyskowski A., Jaremko Ł., Jaremko M. (2021). Genetic and Molecular Factors Determining Grain Weight in Rice. Front. Plant Sci..

[12] Zhang G., Yang Z., Zhou S., Zhu J., Liu X., Luo J. (2024). Cellulose synthase-like *OsCSLD4*: A key regulator of agronomic traits, disease resistance, and metabolic indices in rice. Plant Cell Rep..

[13] Xu X., Ren Y., Wang C., Zhang H., Wang F., Chen J., Liu X., Zheng T., Cai M., Zeng Z. (2019). *OsVIN2* encodes a vacuolar acid invertase that affects grain size by altering sugar metabolism in rice. Plant Cell Rep..

[14] Deng X., Han X., Yu S., Liu Z., Guo D., He Y., Li W., Tao Y., Sun C., Xu P. (2020). *OsINV3* and Its Homolog, *OsINV2*, Control Grain Size in Rice. Int. J. Mol. Sci..

[15] Morey S.R., Hirose T., Hashida Y., Miyao A., Hirochika H., Ohsugi R., Yamagishi J., Aoki N. (2018). Genetic Evidence for the Role of a Rice Vacuolar Invertase as a Molecular Sink Strength Determinant. Rice.

[16] Zhang A., Gao Y., Li Y., Ruan B., Yang S., Liu C., Zhang B., Jiang H., Fang G., Ding S. (2020). Genetic Analysis for Cooking and Eating Quality of Super Rice and Fine Mapping of a Novel Locus *qGC10* for Gel Consistency. Front. Plant Sci..

[17] Wang J., Wang J., Huang L., Kan L., Wang C., Xiong M., Zhou P., Zhou L., Chen C., Zhao D. (2024). ABA-mediated regulation of rice grain quality and seed dormancy via the NF-YB1-SLRL2-bHLH144 Module. Nat. Commun..

[18] Long C., Du Y., Zeng M., Deng X., Zhang Z., Liu D., Zeng Y. (2024). Relationship between Chalkiness and the Structural and Physicochemical Properties of Rice Starch at Different Nighttime Temperatures during the Early Grain-Filling Stage. Foods.

[19] Zhang J., Zheng J., Xie H., Luo J., Huang X., Deng Z., Wu L. (2001). Breeding the new rice restorer line Minghui 86 and its hybrid combinations. J. Plant Genet. Resour..

[20] Xie H., Wu F., Zhang J., Xie H. (2013). Application of indica restorer line Minghui86 for super hybrid Rice. Fujian J. Agric. Sci..

[21] Ma T., Wang Q., Wu T., Hao Q., Huang Y., Mou C., Miao R., Lan J., Zhang F., Wang P. (2025). *Quiescin Sulfhydryl Oxidase-Like 1* Positively Regulates Seed Dormancy in Rice. Plant Biotechnol. J..

[22] Liu H., Ding Y., Zhou Y., Jin W., Xie K., Chen L. (2017). CRISPR-P 2.0: An Improved CRISPR-Cas9 Tool for Genome Editing in Plants. Mol. Plant.

[23] Ma X., Zhang Q., Zhu Q., Liu W., Chen Y., Qiu R., Wang B., Yang Z., Li H., Lin Y. (2015). A Robust CRISPR/Cas9 System for Convenient, High-Efficiency Multiplex Genome Editing in Monocot and Dicot Plants. Mol. Plant.

[24] Hiei Y., Ohta S., Komari T., Kumashiro T. (1994). Efficient transformation of rice (*Oryza sativa* L.) mediated by Agrobacterium and sequence analysis of the boundaries of the T-DNA. Plant J..

[25] Allen G.C., Flores-Vergara M.A., Krasynanski S., Kumar S., Thompson W.F. (2006). A modified protocol for rapid DNA isolation from plant tissues using cetyltrimethylammonium bromide. Nat. Protoc..

[26] Wang L., Li X., Lian H., Ni D., He Y., Chen X., Ruan Y. (2010). Evidence that high activity of vacuolar invertase is required for cotton fiber and Arabidopsis root elongation through osmotic dependent and independent pathways, respectively. Plant Physiol..

