# Supplementary material for: Generation of Novel High-Quality Small-Grained Rice Germplasm by Targeting the OsVIN2 Gene
Source: Biology (Basel). 2025 Dec 30;15(1):64. doi: 10.3390/biology15010064 (PMC12784667; doi:10.3390/biology15010064)
Supplement: Supplementary file 1 [file biology-15-00064-s001.zip › Supplemental Figure S1.pptx]

## Slide 1
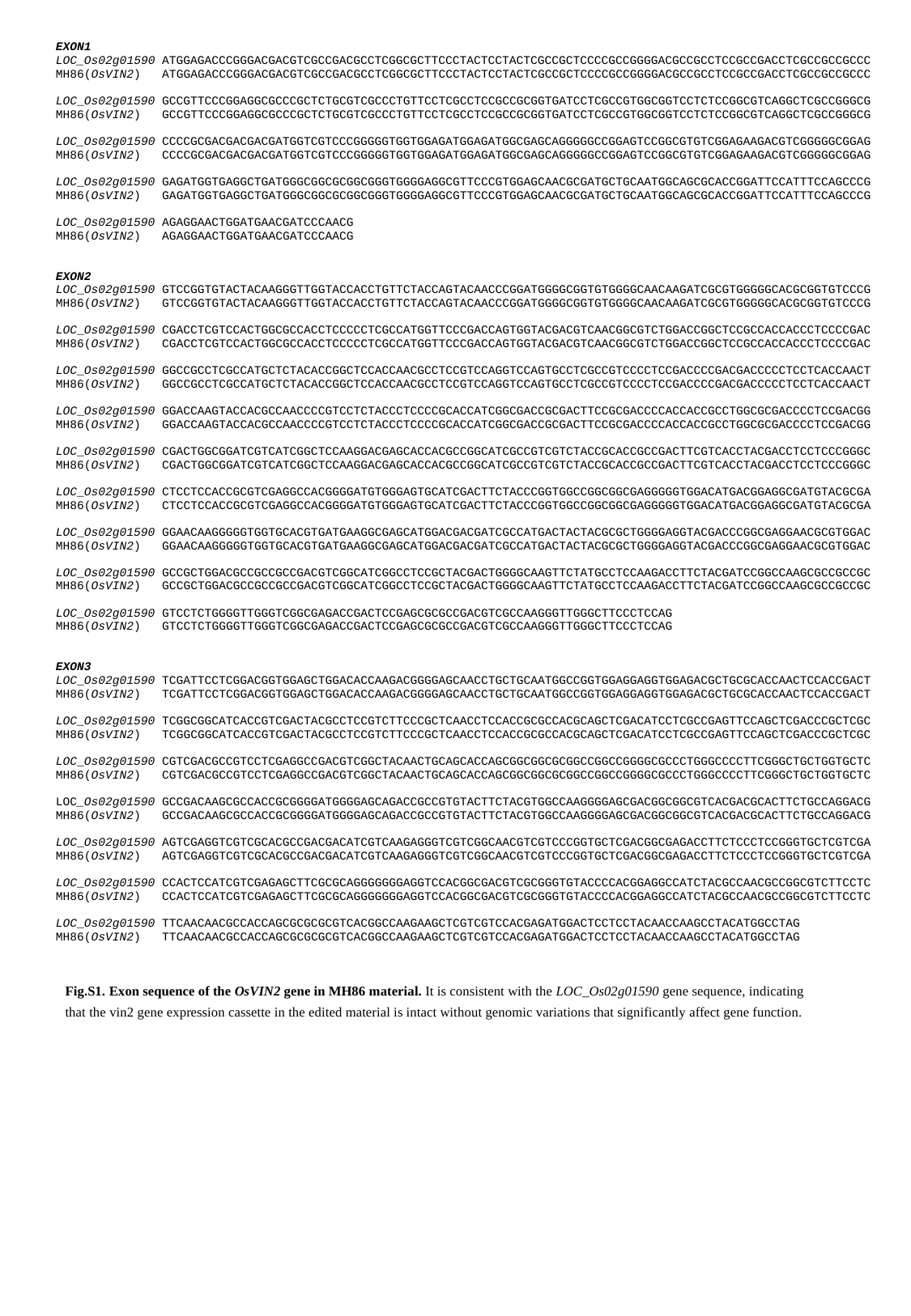

EXON1
LOC_Os02g01590 ATGGAGACCCGGGACGACGTCGCCGACGCCTCGGCGCTTCCCTACTCCTACTCGCCGCTCCCCGCCGGGGACGCCGCCTCCGCCGACCTCGCCGCCGCCC
MH86(OsVIN2) ATGGAGACCCGGGACGACGTCGCCGACGCCTCGGCGCTTCCCTACTCCTACTCGCCGCTCCCCGCCGGGGACGCCGCCTCCGCCGACCTCGCCGCCGCCC
LOC_Os02g01590 GCCGTTCCCGGAGGCGCCCGCTCTGCGTCGCCCTGTTCCTCGCCTCCGCCGCGGTGATCCTCGCCGTGGCGGTCCTCTCCGGCGTCAGGCTCGCCGGGCG
MH86(OsVIN2) GCCGTTCCCGGAGGCGCCCGCTCTGCGTCGCCCTGTTCCTCGCCTCCGCCGCGGTGATCCTCGCCGTGGCGGTCCTCTCCGGCGTCAGGCTCGCCGGGCG
LOC_Os02g01590 CCCCGCGACGACGACGATGGTCGTCCCGGGGGTGGTGGAGATGGAGATGGCGAGCAGGGGGCCGGAGTCCGGCGTGTCGGAGAAGACGTCGGGGGCGGAG
MH86(OsVIN2) CCCCGCGACGACGACGATGGTCGTCCCGGGGGTGGTGGAGATGGAGATGGCGAGCAGGGGGCCGGAGTCCGGCGTGTCGGAGAAGACGTCGGGGGCGGAG
LOC_Os02g01590 GAGATGGTGAGGCTGATGGGCGGCGCGGCGGGTGGGGAGGCGTTCCCGTGGAGCAACGCGATGCTGCAATGGCAGCGCACCGGATTCCATTTCCAGCCCG
MH86(OsVIN2) GAGATGGTGAGGCTGATGGGCGGCGCGGCGGGTGGGGAGGCGTTCCCGTGGAGCAACGCGATGCTGCAATGGCAGCGCACCGGATTCCATTTCCAGCCCG
LOC_Os02g01590 AGAGGAACTGGATGAACGATCCCAACG
MH86(OsVIN2) AGAGGAACTGGATGAACGATCCCAACG
EXON2
LOC_Os02g01590 GTCCGGTGTACTACAAGGGTTGGTACCACCTGTTCTACCAGTACAACCCGGATGGGGCGGTGTGGGGCAACAAGATCGCGTGGGGGCACGCGGTGTCCCG
MH86(OsVIN2) GTCCGGTGTACTACAAGGGTTGGTACCACCTGTTCTACCAGTACAACCCGGATGGGGCGGTGTGGGGCAACAAGATCGCGTGGGGGCACGCGGTGTCCCG
LOC_Os02g01590 CGACCTCGTCCACTGGCGCCACCTCCCCCTCGCCATGGTTCCCGACCAGTGGTACGACGTCAACGGCGTCTGGACCGGCTCCGCCACCACCCTCCCCGAC
MH86(OsVIN2) CGACCTCGTCCACTGGCGCCACCTCCCCCTCGCCATGGTTCCCGACCAGTGGTACGACGTCAACGGCGTCTGGACCGGCTCCGCCACCACCCTCCCCGAC
LOC_Os02g01590 GGCCGCCTCGCCATGCTCTACACCGGCTCCACCAACGCCTCCGTCCAGGTCCAGTGCCTCGCCGTCCCCTCCGACCCCGACGACCCCCTCCTCACCAACT
MH86(OsVIN2) GGCCGCCTCGCCATGCTCTACACCGGCTCCACCAACGCCTCCGTCCAGGTCCAGTGCCTCGCCGTCCCCTCCGACCCCGACGACCCCCTCCTCACCAACT
LOC_Os02g01590 GGACCAAGTACCACGCCAACCCCGTCCTCTACCCTCCCCGCACCATCGGCGACCGCGACTTCCGCGACCCCACCACCGCCTGGCGCGACCCCTCCGACGG
MH86(OsVIN2) GGACCAAGTACCACGCCAACCCCGTCCTCTACCCTCCCCGCACCATCGGCGACCGCGACTTCCGCGACCCCACCACCGCCTGGCGCGACCCCTCCGACGG
LOC_Os02g01590 CGACTGGCGGATCGTCATCGGCTCCAAGGACGAGCACCACGCCGGCATCGCCGTCGTCTACCGCACCGCCGACTTCGTCACCTACGACCTCCTCCCGGGC
MH86(OsVIN2) CGACTGGCGGATCGTCATCGGCTCCAAGGACGAGCACCACGCCGGCATCGCCGTCGTCTACCGCACCGCCGACTTCGTCACCTACGACCTCCTCCCGGGC
LOC_Os02g01590 CTCCTCCACCGCGTCGAGGCCACGGGGATGTGGGAGTGCATCGACTTCTACCCGGTGGCCGGCGGCGAGGGGGTGGACATGACGGAGGCGATGTACGCGA
MH86(OsVIN2) CTCCTCCACCGCGTCGAGGCCACGGGGATGTGGGAGTGCATCGACTTCTACCCGGTGGCCGGCGGCGAGGGGGTGGACATGACGGAGGCGATGTACGCGA
LOC_Os02g01590 GGAACAAGGGGGTGGTGCACGTGATGAAGGCGAGCATGGACGACGATCGCCATGACTACTACGCGCTGGGGAGGTACGACCCGGCGAGGAACGCGTGGAC
MH86(OsVIN2) GGAACAAGGGGGTGGTGCACGTGATGAAGGCGAGCATGGACGACGATCGCCATGACTACTACGCGCTGGGGAGGTACGACCCGGCGAGGAACGCGTGGAC
LOC_Os02g01590 GCCGCTGGACGCCGCCGCCGACGTCGGCATCGGCCTCCGCTACGACTGGGGCAAGTTCTATGCCTCCAAGACCTTCTACGATCCGGCCAAGCGCCGCCGC
MH86(OsVIN2) GCCGCTGGACGCCGCCGCCGACGTCGGCATCGGCCTCCGCTACGACTGGGGCAAGTTCTATGCCTCCAAGACCTTCTACGATCCGGCCAAGCGCCGCCGC
LOC_Os02g01590 GTCCTCTGGGGTTGGGTCGGCGAGACCGACTCCGAGCGCGCCGACGTCGCCAAGGGTTGGGCTTCCCTCCAG
MH86(OsVIN2) GTCCTCTGGGGTTGGGTCGGCGAGACCGACTCCGAGCGCGCCGACGTCGCCAAGGGTTGGGCTTCCCTCCAG
EXON3
LOC_Os02g01590 TCGATTCCTCGGACGGTGGAGCTGGACACCAAGACGGGGAGCAACCTGCTGCAATGGCCGGTGGAGGAGGTGGAGACGCTGCGCACCAACTCCACCGACT
MH86(OsVIN2) TCGATTCCTCGGACGGTGGAGCTGGACACCAAGACGGGGAGCAACCTGCTGCAATGGCCGGTGGAGGAGGTGGAGACGCTGCGCACCAACTCCACCGACT
LOC_Os02g01590 TCGGCGGCATCACCGTCGACTACGCCTCCGTCTTCCCGCTCAACCTCCACCGCGCCACGCAGCTCGACATCCTCGCCGAGTTCCAGCTCGACCCGCTCGC
MH86(OsVIN2) TCGGCGGCATCACCGTCGACTACGCCTCCGTCTTCCCGCTCAACCTCCACCGCGCCACGCAGCTCGACATCCTCGCCGAGTTCCAGCTCGACCCGCTCGC
LOC_Os02g01590 CGTCGACGCCGTCCTCGAGGCCGACGTCGGCTACAACTGCAGCACCAGCGGCGGCGCGGCCGGCCGGGGCGCCCTGGGCCCCTTCGGGCTGCTGGTGCTC
MH86(OsVIN2) CGTCGACGCCGTCCTCGAGGCCGACGTCGGCTACAACTGCAGCACCAGCGGCGGCGCGGCCGGCCGGGGCGCCCTGGGCCCCTTCGGGCTGCTGGTGCTC
LOC_Os02g01590 GCCGACAAGCGCCACCGCGGGGATGGGGAGCAGACCGCCGTGTACTTCTACGTGGCCAAGGGGAGCGACGGCGGCGTCACGACGCACTTCTGCCAGGACG
MH86(OsVIN2) GCCGACAAGCGCCACCGCGGGGATGGGGAGCAGACCGCCGTGTACTTCTACGTGGCCAAGGGGAGCGACGGCGGCGTCACGACGCACTTCTGCCAGGACG
LOC_Os02g01590 AGTCGAGGTCGTCGCACGCCGACGACATCGTCAAGAGGGTCGTCGGCAACGTCGTCCCGGTGCTCGACGGCGAGACCTTCTCCCTCCGGGTGCTCGTCGA
MH86(OsVIN2) AGTCGAGGTCGTCGCACGCCGACGACATCGTCAAGAGGGTCGTCGGCAACGTCGTCCCGGTGCTCGACGGCGAGACCTTCTCCCTCCGGGTGCTCGTCGA
LOC_Os02g01590 CCACTCCATCGTCGAGAGCTTCGCGCAGGGGGGGAGGTCCACGGCGACGTCGCGGGTGTACCCCACGGAGGCCATCTACGCCAACGCCGGCGTCTTCCTC
MH86(OsVIN2) CCACTCCATCGTCGAGAGCTTCGCGCAGGGGGGGAGGTCCACGGCGACGTCGCGGGTGTACCCCACGGAGGCCATCTACGCCAACGCCGGCGTCTTCCTC
LOC_Os02g01590 TTCAACAACGCCACCAGCGCGCGCGTCACGGCCAAGAAGCTCGTCGTCCACGAGATGGACTCCTCCTACAACCAAGCCTACATGGCCTAG
MH86(OsVIN2) TTCAACAACGCCACCAGCGCGCGCGTCACGGCCAAGAAGCTCGTCGTCCACGAGATGGACTCCTCCTACAACCAAGCCTACATGGCCTAG
Fig.S1. Exon sequence of the OsVIN2 gene in MH86 material. It is consistent with the LOC_Os02g01590 gene sequence, indicating that the vin2 gene expression cassette in the edited material is intact without genomic variations that significantly affect gene function.
