# Supplementary material for: Generation of Novel High-Quality Small-Grained Rice Germplasm by Targeting the OsVIN2 Gene
Source: Biology (Basel). 2025 Dec 30;15(1):64. doi: 10.3390/biology15010064 (PMC12784667; doi:10.3390/biology15010064)
Supplement: Supplementary file 1 [file biology-15-00064-s001.zip › Supplemental Figure S2.pptx]

## Slide 1
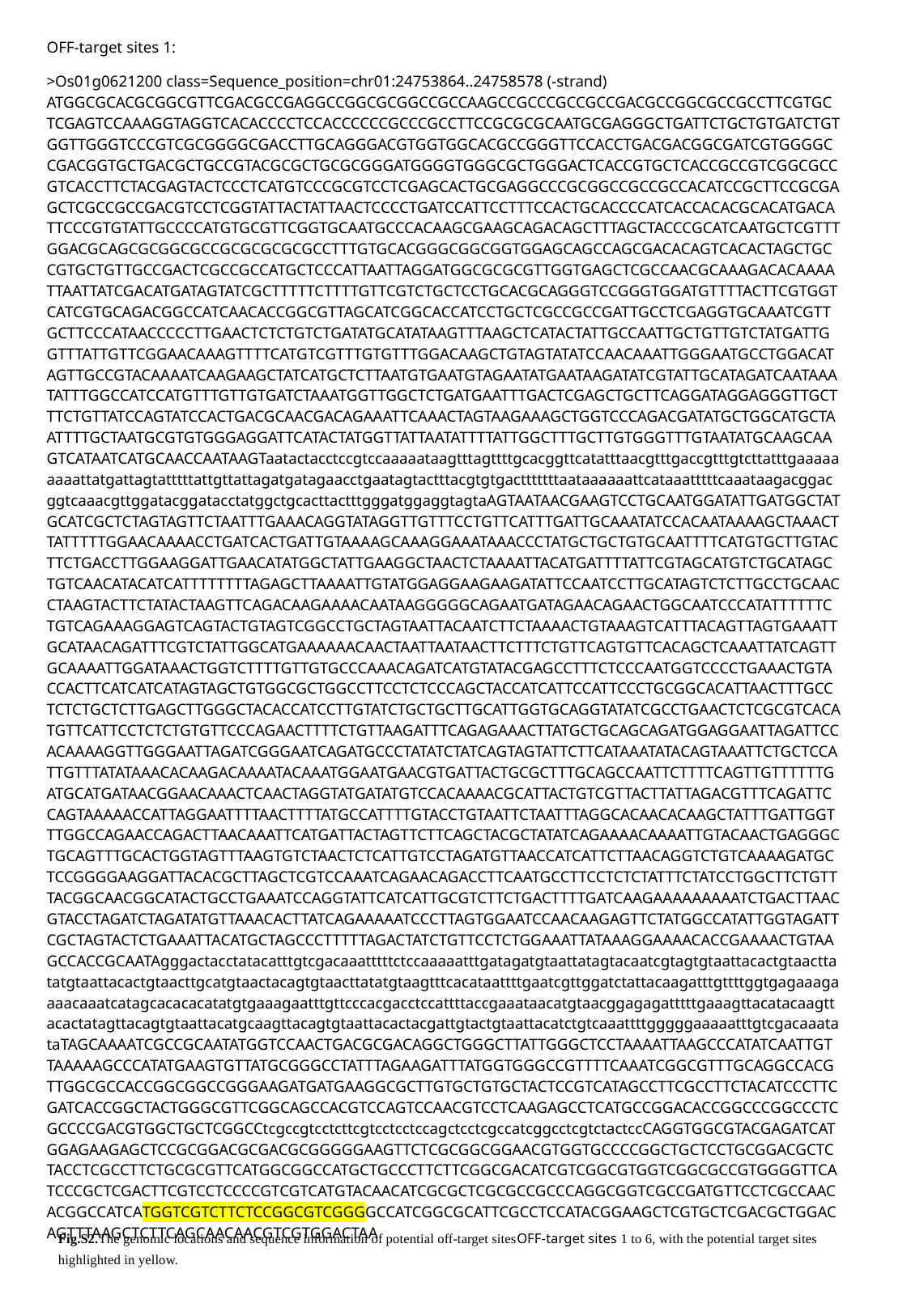

OFF-target sites 1:
>Os01g0621200 class=Sequence_position=chr01:24753864..24758578 (-strand) ATGGCGCACGCGGCGTTCGACGCCGAGGCCGGCGCGGCCGCCAAGCCGCCCGCCGCCGACGCCGGCGCCGCCTTCGTGCTCGAGTCCAAAGGTAGGTCACACCCCTCCACCCCCCGCCCGCCTTCCGCGCGCAATGCGAGGGCTGATTCTGCTGTGATCTGTGGTTGGGTCCCGTCGCGGGGCGACCTTGCAGGGACGTGGTGGCACGCCGGGTTCCACCTGACGACGGCGATCGTGGGGCCGACGGTGCTGACGCTGCCGTACGCGCTGCGCGGGATGGGGTGGGCGCTGGGACTCACCGTGCTCACCGCCGTCGGCGCCGTCACCTTCTACGAGTACTCCCTCATGTCCCGCGTCCTCGAGCACTGCGAGGCCCGCGGCCGCCGCCACATCCGCTTCCGCGAGCTCGCCGCCGACGTCCTCGGTATTACTATTAACTCCCCTGATCCATTCCTTTCCACTGCACCCCATCACCACACGCACATGACATTCCCGTGTATTGCCCCATGTGCGTTCGGTGCAATGCCCACAAGCGAAGCAGACAGCTTTAGCTACCCGCATCAATGCTCGTTTGGACGCAGCGCGGCGCCGCGCGCGCGCCTTTGTGCACGGGCGGCGGTGGAGCAGCCAGCGACACAGTCACACTAGCTGCCGTGCTGTTGCCGACTCGCCGCCATGCTCCCATTAATTAGGATGGCGCGCGTTGGTGAGCTCGCCAACGCAAAGACACAAAATTAATTATCGACATGATAGTATCGCTTTTTCTTTTGTTCGTCTGCTCCTGCACGCAGGGTCCGGGTGGATGTTTTACTTCGTGGTCATCGTGCAGACGGCCATCAACACCGGCGTTAGCATCGGCACCATCCTGCTCGCCGCCGATTGCCTCGAGGTGCAAATCGTTGCTTCCCATAACCCCCTTGAACTCTCTGTCTGATATGCATATAAGTTTAAGCTCATACTATTGCCAATTGCTGTTGTCTATGATTGGTTTATTGTTCGGAACAAAGTTTTCATGTCGTTTGTGTTTGGACAAGCTGTAGTATATCCAACAAATTGGGAATGCCTGGACATAGTTGCCGTACAAAATCAAGAAGCTATCATGCTCTTAATGTGAATGTAGAATATGAATAAGATATCGTATTGCATAGATCAATAAATATTTGGCCATCCATGTTTGTTGTGATCTAAATGGTTGGCTCTGATGAATTTGACTCGAGCTGCTTCAGGATAGGAGGGTTGCTTTCTGTTATCCAGTATCCACTGACGCAACGACAGAAATTCAAACTAGTAAGAAAGCTGGTCCCAGACGATATGCTGGCATGCTAATTTTGCTAATGCGTGTGGGAGGATTCATACTATGGTTATTAATATTTTATTGGCTTTGCTTGTGGGTTTGTAATATGCAAGCAAGTCATAATCATGCAACCAATAAGTaatactacctccgtccaaaaataagtttagttttgcacggttcatatttaacgtttgaccgtttgtcttatttgaaaaaaaaattatgattagtatttttattgttattagatgatagaacctgaatagtactttacgtgtgactttttttaataaaaaattcataaatttttcaaataagacggacggtcaaacgttggatacggatacctatggctgcacttactttgggatggaggtagtaAGTAATAACGAAGTCCTGCAATGGATATTGATGGCTATGCATCGCTCTAGTAGTTCTAATTTGAAACAGGTATAGGTTGTTTCCTGTTCATTTGATTGCAAATATCCACAATAAAAGCTAAACTTATTTTTGGAACAAAACCTGATCACTGATTGTAAAAGCAAAGGAAATAAACCCTATGCTGCTGTGCAATTTTCATGTGCTTGTACTTCTGACCTTGGAAGGATTGAACATATGGCTATTGAAGGCTAACTCTAAAATTACATGATTTTATTCGTAGCATGTCTGCATAGCTGTCAACATACATCATTTTTTTTAGAGCTTAAAATTGTATGGAGGAAGAAGATATTCCAATCCTTGCATAGTCTCTTGCCTGCAACCTAAGTACTTCTATACTAAGTTCAGACAAGAAAACAATAAGGGGGCAGAATGATAGAACAGAACTGGCAATCCCATATTTTTTCTGTCAGAAAGGAGTCAGTACTGTAGTCGGCCTGCTAGTAATTACAATCTTCTAAAACTGTAAAGTCATTTACAGTTAGTGAAATTGCATAACAGATTTCGTCTATTGGCATGAAAAAACAACTAATTAATAACTTCTTTCTGTTCAGTGTTCACAGCTCAAATTATCAGTTGCAAAATTGGATAAACTGGTCTTTTGTTGTGCCCAAACAGATCATGTATACGAGCCTTTCTCCCAATGGTCCCCTGAAACTGTACCACTTCATCATCATAGTAGCTGTGGCGCTGGCCTTCCTCTCCCAGCTACCATCATTCCATTCCCTGCGGCACATTAACTTTGCCTCTCTGCTCTTGAGCTTGGGCTACACCATCCTTGTATCTGCTGCTTGCATTGGTGCAGGTATATCGCCTGAACTCTCGCGTCACATGTTCATTCCTCTCTGTGTTCCCAGAACTTTTCTGTTAAGATTTCAGAGAAACTTATGCTGCAGCAGATGGAGGAATTAGATTCCACAAAAGGTTGGGAATTAGATCGGGAATCAGATGCCCTATATCTATCAGTAGTATTCTTCATAAATATACAGTAAATTCTGCTCCATTGTTTATATAAACACAAGACAAAATACAAATGGAATGAACGTGATTACTGCGCTTTGCAGCCAATTCTTTTCAGTTGTTTTTTGATGCATGATAACGGAACAAACTCAACTAGGTATGATATGTCCACAAAACGCATTACTGTCGTTACTTATTAGACGTTTCAGATTCCAGTAAAAACCATTAGGAATTTTAACTTTTATGCCATTTTGTACCTGTAATTCTAATTTAGGCACAACACAAGCTATTTGATTGGTTTGGCCAGAACCAGACTTAACAAATTCATGATTACTAGTTCTTCAGCTACGCTATATCAGAAAACAAAATTGTACAACTGAGGGCTGCAGTTTGCACTGGTAGTTTAAGTGTCTAACTCTCATTGTCCTAGATGTTAACCATCATTCTTAACAGGTCTGTCAAAAGATGCTCCGGGGAAGGATTACACGCTTAGCTCGTCCAAATCAGAACAGACCTTCAATGCCTTCCTCTCTATTTCTATCCTGGCTTCTGTTTACGGCAACGGCATACTGCCTGAAATCCAGGTATTCATCATTGCGTCTTCTGACTTTTGATCAAGAAAAAAAAATCTGACTTAACGTACCTAGATCTAGATATGTTAAACACTTATCAGAAAAATCCCTTAGTGGAATCCAACAAGAGTTCTATGGCCATATTGGTAGATTCGCTAGTACTCTGAAATTACATGCTAGCCCTTTTTAGACTATCTGTTCCTCTGGAAATTATAAAGGAAAACACCGAAAACTGTAAGCCACCGCAATAgggactacctatacatttgtcgacaaatttttctccaaaaatttgatagatgtaattatagtacaatcgtagtgtaattacactgtaacttatatgtaattacactgtaacttgcatgtaactacagtgtaacttatatgtaagtttcacataattttgaatcgttggatctattacaagatttgttttggtgagaaagaaaacaaatcatagcacacacatatgtgaaagaatttgttcccacgacctccattttaccgaaataacatgtaacggagagatttttgaaagttacatacaagttacactatagttacagtgtaattacatgcaagttacagtgtaattacactacgattgtactgtaattacatctgtcaaattttgggggaaaaatttgtcgacaaatataTAGCAAAATCGCCGCAATATGGTCCAACTGACGCGACAGGCTGGGCTTATTGGGCTCCTAAAATTAAGCCCATATCAATTGTTAAAAAGCCCATATGAAGTGTTATGCGGGCCTATTTAGAAGATTTATGGTGGGCCGTTTTCAAATCGGCGTTTGCAGGCCACGTTGGCGCCACCGGCGGCCGGGAAGATGATGAAGGCGCTTGTGCTGTGCTACTCCGTCATAGCCTTCGCCTTCTACATCCCTTCGATCACCGGCTACTGGGCGTTCGGCAGCCACGTCCAGTCCAACGTCCTCAAGAGCCTCATGCCGGACACCGGCCCGGCCCTCGCCCCGACGTGGCTGCTCGGCCtcgccgtcctcttcgtcctcctccagctcctcgccatcggcctcgtctactccCAGGTGGCGTACGAGATCATGGAGAAGAGCTCCGCGGACGCGACGCGGGGGAAGTTCTCGCGGCGGAACGTGGTGCCCCGGCTGCTCCTGCGGACGCTCTACCTCGCCTTCTGCGCGTTCATGGCGGCCATGCTGCCCTTCTTCGGCGACATCGTCGGCGTGGTCGGCGCCGTGGGGTTCATCCCGCTCGACTTCGTCCTCCCCGTCGTCATGTACAACATCGCGCTCGCGCCGCCCAGGCGGTCGCCGATGTTCCTCGCCAACACGGCCATCATGGTCGTCTTCTCCGGCGTCGGGGCCATCGGCGCATTCGCCTCCATACGGAAGCTCGTGCTCGACGCTGGACAGTTTAAGCTCTTCAGCAACAACGTCGTGGACTAA
Fig.S2.The genomic locations and sequence information of potential off-target sitesOFF-target sites 1 to 6, with the potential target sites highlighted in yellow.

## Slide 2
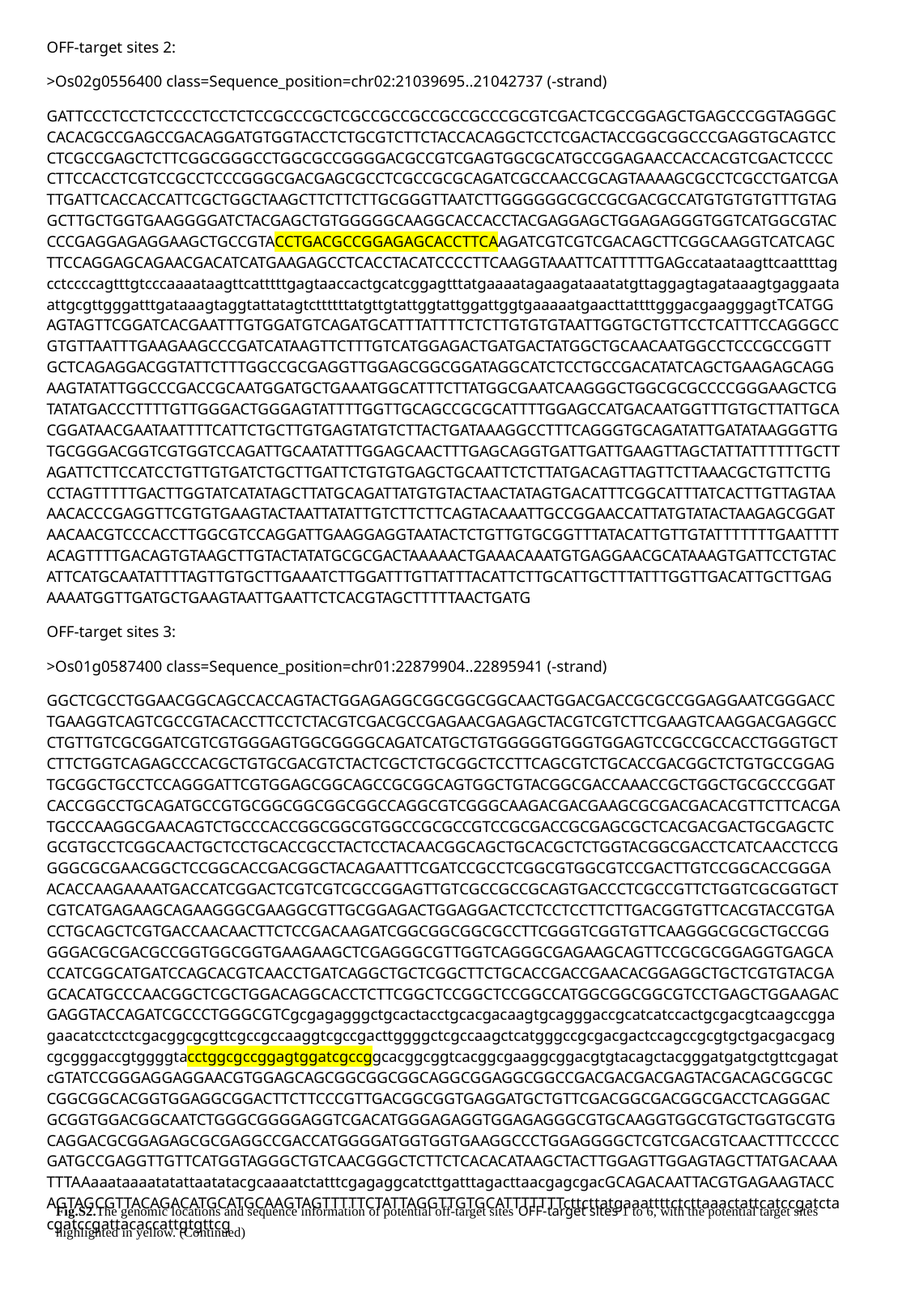

OFF-target sites 2:
>Os02g0556400 class=Sequence_position=chr02:21039695..21042737 (-strand)
GATTCCCTCCTCTCCCCTCCTCTCCGCCCGCTCGCCGCCGCCGCCGCCCGCGTCGACTCGCCGGAGCTGAGCCCGGTAGGGCCACACGCCGAGCCGACAGGATGTGGTACCTCTGCGTCTTCTACCACAGGCTCCTCGACTACCGGCGGCCCGAGGTGCAGTCCCTCGCCGAGCTCTTCGGCGGGCCTGGCGCCGGGGACGCCGTCGAGTGGCGCATGCCGGAGAACCACCACGTCGACTCCCCCTTCCACCTCGTCCGCCTCCCGGGCGACGAGCGCCTCGCCGCGCAGATCGCCAACCGCAGTAAAAGCGCCTCGCCTGATCGATTGATTCACCACCATTCGCTGGCTAAGCTTCTTCTTGCGGGTTAATCTTGGGGGGCGCCGCGACGCCATGTGTGTGTTTGTAGGCTTGCTGGTGAAGGGGATCTACGAGCTGTGGGGGCAAGGCACCACCTACGAGGAGCTGGAGAGGGTGGTCATGGCGTACCCCGAGGAGAGGAAGCTGCCGTACCTGACGCCGGAGAGCACCTTCAAGATCGTCGTCGACAGCTTCGGCAAGGTCATCAGCTTCCAGGAGCAGAACGACATCATGAAGAGCCTCACCTACATCCCCTTCAAGGTAAATTCATTTTTGAGccataataagttcaattttagcctccccagtttgtcccaaaataagttcatttttgagtaaccactgcatcggagtttatgaaaatagaagataaatatgttaggagtagataaagtgaggaataattgcgttgggatttgataaagtaggtattatagtcttttttatgttgtattggtattggattggtgaaaaatgaacttattttgggacgaagggagtTCATGGAGTAGTTCGGATCACGAATTTGTGGATGTCAGATGCATTTATTTTCTCTTGTGTGTAATTGGTGCTGTTCCTCATTTCCAGGGCCGTGTTAATTTGAAGAAGCCCGATCATAAGTTCTTTGTCATGGAGACTGATGACTATGGCTGCAACAATGGCCTCCCGCCGGTTGCTCAGAGGACGGTATTCTTTGGCCGCGAGGTTGGAGCGGCGGATAGGCATCTCCTGCCGACATATCAGCTGAAGAGCAGGAAGTATATTGGCCCGACCGCAATGGATGCTGAAATGGCATTTCTTATGGCGAATCAAGGGCTGGCGCGCCCCGGGAAGCTCGTATATGACCCTTTTGTTGGGACTGGGAGTATTTTGGTTGCAGCCGCGCATTTTGGAGCCATGACAATGGTTTGTGCTTATTGCACGGATAACGAATAATTTTCATTCTGCTTGTGAGTATGTCTTACTGATAAAGGCCTTTCAGGGTGCAGATATTGATATAAGGGTTGTGCGGGACGGTCGTGGTCCAGATTGCAATATTTGGAGCAACTTTGAGCAGGTGATTGATTGAAGTTAGCTATTATTTTTTGCTTAGATTCTTCCATCCTGTTGTGATCTGCTTGATTCTGTGTGAGCTGCAATTCTCTTATGACAGTTAGTTCTTAAACGCTGTTCTTGCCTAGTTTTTGACTTGGTATCATATAGCTTATGCAGATTATGTGTACTAACTATAGTGACATTTCGGCATTTATCACTTGTTAGTAAAACACCCGAGGTTCGTGTGAAGTACTAATTATATTGTCTTCTTCAGTACAAATTGCCGGAACCATTATGTATACTAAGAGCGGATAACAACGTCCCACCTTGGCGTCCAGGATTGAAGGAGGTAATACTCTGTTGTGCGGTTTATACATTGTTGTATTTTTTTGAATTTTACAGTTTTGACAGTGTAAGCTTGTACTATATGCGCGACTAAAAACTGAAACAAATGTGAGGAACGCATAAAGTGATTCCTGTACATTCATGCAATATTTTAGTTGTGCTTGAAATCTTGGATTTGTTATTTACATTCTTGCATTGCTTTATTTGGTTGACATTGCTTGAGAAAATGGTTGATGCTGAAGTAATTGAATTCTCACGTAGCTTTTTAACTGATG
OFF-target sites 3:
>Os01g0587400 class=Sequence_position=chr01:22879904..22895941 (-strand)
GGCTCGCCTGGAACGGCAGCCACCAGTACTGGAGAGGCGGCGGCGGCAACTGGACGACCGCGCCGGAGGAATCGGGACCTGAAGGTCAGTCGCCGTACACCTTCCTCTACGTCGACGCCGAGAACGAGAGCTACGTCGTCTTCGAAGTCAAGGACGAGGCCCTGTTGTCGCGGATCGTCGTGGGAGTGGCGGGGCAGATCATGCTGTGGGGGTGGGTGGAGTCCGCCGCCACCTGGGTGCTCTTCTGGTCAGAGCCCACGCTGTGCGACGTCTACTCGCTCTGCGGCTCCTTCAGCGTCTGCACCGACGGCTCTGTGCCGGAGTGCGGCTGCCTCCAGGGATTCGTGGAGCGGCAGCCGCGGCAGTGGCTGTACGGCGACCAAACCGCTGGCTGCGCCCGGATCACCGGCCTGCAGATGCCGTGCGGCGGCGGCGGCCAGGCGTCGGGCAAGACGACGAAGCGCGACGACACGTTCTTCACGATGCCCAAGGCGAACAGTCTGCCCACCGGCGGCGTGGCCGCGCCGTCCGCGACCGCGAGCGCTCACGACGACTGCGAGCTCGCGTGCCTCGGCAACTGCTCCTGCACCGCCTACTCCTACAACGGCAGCTGCACGCTCTGGTACGGCGACCTCATCAACCTCCGGGGCGCGAACGGCTCCGGCACCGACGGCTACAGAATTTCGATCCGCCTCGGCGTGGCGTCCGACTTGTCCGGCACCGGGAACACCAAGAAAATGACCATCGGACTCGTCGTCGCCGGAGTTGTCGCCGCCGCAGTGACCCTCGCCGTTCTGGTCGCGGTGCTCGTCATGAGAAGCAGAAGGGCGAAGGCGTTGCGGAGACTGGAGGACTCCTCCTCCTTCTTGACGGTGTTCACGTACCGTGACCTGCAGCTCGTGACCAACAACTTCTCCGACAAGATCGGCGGCGGCGCCTTCGGGTCGGTGTTCAAGGGCGCGCTGCCGGGGGACGCGACGCCGGTGGCGGTGAAGAAGCTCGAGGGCGTTGGTCAGGGCGAGAAGCAGTTCCGCGCGGAGGTGAGCACCATCGGCATGATCCAGCACGTCAACCTGATCAGGCTGCTCGGCTTCTGCACCGACCGAACACGGAGGCTGCTCGTGTACGAGCACATGCCCAACGGCTCGCTGGACAGGCACCTCTTCGGCTCCGGCTCCGGCCATGGCGGCGGCGTCCTGAGCTGGAAGACGAGGTACCAGATCGCCCTGGGCGTCgcgagagggctgcactacctgcacgacaagtgcagggaccgcatcatccactgcgacgtcaagccggagaacatcctcctcgacggcgcgttcgccgccaaggtcgccgacttggggctcgccaagctcatgggccgcgacgactccagccgcgtgctgacgacgacgcgcgggaccgtggggtacctggcgccggagtggatcgccggcacggcggtcacggcgaaggcggacgtgtacagctacgggatgatgctgttcgagatcGTATCCGGGAGGAGGAACGTGGAGCAGCGGCGGCGGCAGGCGGAGGCGGCCGACGACGACGAGTACGACAGCGGCGCCGGCGGCACGGTGGAGGCGGACTTCTTCCCGTTGACGGCGGTGAGGATGCTGTTCGACGGCGACGGCGACCTCAGGGACGCGGTGGACGGCAATCTGGGCGGGGAGGTCGACATGGGAGAGGTGGAGAGGGCGTGCAAGGTGGCGTGCTGGTGCGTGCAGGACGCGGAGAGCGCGAGGCCGACCATGGGGATGGTGGTGAAGGCCCTGGAGGGGCTCGTCGACGTCAACTTTCCCCCGATGCCGAGGTTGTTCATGGTAGGGCTGTCAACGGGCTCTTCTCACACATAAGCTACTTGGAGTTGGAGTAGCTTATGACAAATTTAAaaataaaatatattaatatacgcaaaatctatttcgagaggcatcttgatttagacttaacgagcgacGCAGACAATTACGTGAGAAGTACCAGTAGCGTTACAGACATGCATGCAAGTAGTTTTTCTATTAGGTTGTGCATTTTTTTcttcttatgaaattttctcttaaactattcatccgatctacgatccgattacaccattgtgttcg
Fig.S2.The genomic locations and sequence information of potential off-target sites OFF-target sites 1 to 6, with the potential target sites highlighted in yellow. (Continued)

## Slide 3
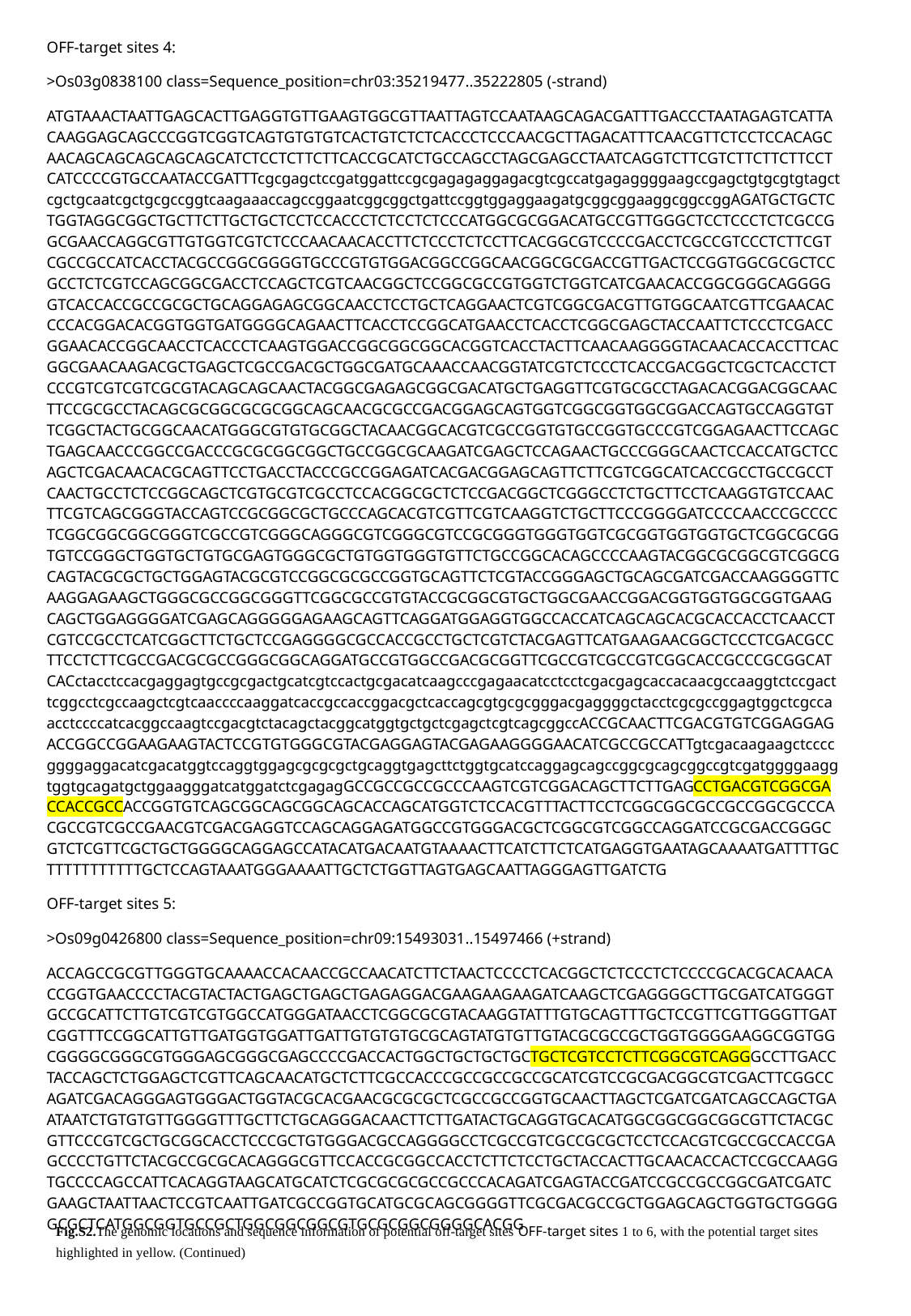

OFF-target sites 4:
>Os03g0838100 class=Sequence_position=chr03:35219477..35222805 (-strand)
ATGTAAACTAATTGAGCACTTGAGGTGTTGAAGTGGCGTTAATTAGTCCAATAAGCAGACGATTTGACCCTAATAGAGTCATTACAAGGAGCAGCCCGGTCGGTCAGTGTGTGTCACTGTCTCTCACCCTCCCAACGCTTAGACATTTCAACGTTCTCCTCCACAGCAACAGCAGCAGCAGCAGCATCTCCTCTTCTTCACCGCATCTGCCAGCCTAGCGAGCCTAATCAGGTCTTCGTCTTCTTCTTCCTCATCCCCGTGCCAATACCGATTTcgcgagctccgatggattccgcgagagaggagacgtcgccatgagaggggaagccgagctgtgcgtgtagctcgctgcaatcgctgcgccggtcaagaaaccagccggaatcggcggctgattccggtggaggaagatgcggcggaaggcggccggAGATGCTGCTCTGGTAGGCGGCTGCTTCTTGCTGCTCCTCCACCCTCTCCTCTCCCATGGCGCGGACATGCCGTTGGGCTCCTCCCTCTCGCCGGCGAACCAGGCGTTGTGGTCGTCTCCCAACAACACCTTCTCCCTCTCCTTCACGGCGTCCCCGACCTCGCCGTCCCTCTTCGTCGCCGCCATCACCTACGCCGGCGGGGTGCCCGTGTGGACGGCCGGCAACGGCGCGACCGTTGACTCCGGTGGCGCGCTCCGCCTCTCGTCCAGCGGCGACCTCCAGCTCGTCAACGGCTCCGGCGCCGTGGTCTGGTCATCGAACACCGGCGGGCAGGGGGTCACCACCGCCGCGCTGCAGGAGAGCGGCAACCTCCTGCTCAGGAACTCGTCGGCGACGTTGTGGCAATCGTTCGAACACCCCACGGACACGGTGGTGATGGGGCAGAACTTCACCTCCGGCATGAACCTCACCTCGGCGAGCTACCAATTCTCCCTCGACCGGAACACCGGCAACCTCACCCTCAAGTGGACCGGCGGCGGCACGGTCACCTACTTCAACAAGGGGTACAACACCACCTTCACGGCGAACAAGACGCTGAGCTCGCCGACGCTGGCGATGCAAACCAACGGTATCGTCTCCCTCACCGACGGCTCGCTCACCTCTCCCGTCGTCGTCGCGTACAGCAGCAACTACGGCGAGAGCGGCGACATGCTGAGGTTCGTGCGCCTAGACACGGACGGCAACTTCCGCGCCTACAGCGCGGCGCGCGGCAGCAACGCGCCGACGGAGCAGTGGTCGGCGGTGGCGGACCAGTGCCAGGTGTTCGGCTACTGCGGCAACATGGGCGTGTGCGGCTACAACGGCACGTCGCCGGTGTGCCGGTGCCCGTCGGAGAACTTCCAGCTGAGCAACCCGGCCGACCCGCGCGGCGGCTGCCGGCGCAAGATCGAGCTCCAGAACTGCCCGGGCAACTCCACCATGCTCCAGCTCGACAACACGCAGTTCCTGACCTACCCGCCGGAGATCACGACGGAGCAGTTCTTCGTCGGCATCACCGCCTGCCGCCTCAACTGCCTCTCCGGCAGCTCGTGCGTCGCCTCCACGGCGCTCTCCGACGGCTCGGGCCTCTGCTTCCTCAAGGTGTCCAACTTCGTCAGCGGGTACCAGTCCGCGGCGCTGCCCAGCACGTCGTTCGTCAAGGTCTGCTTCCCGGGGATCCCCAACCCGCCCCTCGGCGGCGGCGGGTCGCCGTCGGGCAGGGCGTCGGGCGTCCGCGGGTGGGTGGTCGCGGTGGTGGTGCTCGGCGCGGTGTCCGGGCTGGTGCTGTGCGAGTGGGCGCTGTGGTGGGTGTTCTGCCGGCACAGCCCCAAGTACGGCGCGGCGTCGGCGCAGTACGCGCTGCTGGAGTACGCGTCCGGCGCGCCGGTGCAGTTCTCGTACCGGGAGCTGCAGCGATCGACCAAGGGGTTCAAGGAGAAGCTGGGCGCCGGCGGGTTCGGCGCCGTGTACCGCGGCGTGCTGGCGAACCGGACGGTGGTGGCGGTGAAGCAGCTGGAGGGGATCGAGCAGGGGGAGAAGCAGTTCAGGATGGAGGTGGCCACCATCAGCAGCACGCACCACCTCAACCTCGTCCGCCTCATCGGCTTCTGCTCCGAGGGGCGCCACCGCCTGCTCGTCTACGAGTTCATGAAGAACGGCTCCCTCGACGCCTTCCTCTTCGCCGACGCGCCGGGCGGCAGGATGCCGTGGCCGACGCGGTTCGCCGTCGCCGTCGGCACCGCCCGCGGCATCACctacctccacgaggagtgccgcgactgcatcgtccactgcgacatcaagcccgagaacatcctcctcgacgagcaccacaacgccaaggtctccgacttcggcctcgccaagctcgtcaaccccaaggatcaccgccaccggacgctcaccagcgtgcgcgggacgaggggctacctcgcgccggagtggctcgccaacctccccatcacggccaagtccgacgtctacagctacggcatggtgctgctcgagctcgtcagcggccACCGCAACTTCGACGTGTCGGAGGAGACCGGCCGGAAGAAGTACTCCGTGTGGGCGTACGAGGAGTACGAGAAGGGGAACATCGCCGCCATTgtcgacaagaagctccccggggaggacatcgacatggtccaggtggagcgcgcgctgcaggtgagcttctggtgcatccaggagcagccggcgcagcggccgtcgatggggaaggtggtgcagatgctggaagggatcatggatctcgagagGCCGCCGCCGCCCAAGTCGTCGGACAGCTTCTTGAGCCTGACGTCGGCGACCACCGCCACCGGTGTCAGCGGCAGCGGCAGCACCAGCATGGTCTCCACGTTTACTTCCTCGGCGGCGCCGCCGGCGCCCACGCCGTCGCCGAACGTCGACGAGGTCCAGCAGGAGATGGCCGTGGGACGCTCGGCGTCGGCCAGGATCCGCGACCGGGCGTCTCGTTCGCTGCTGGGGCAGGAGCCATACATGACAATGTAAAACTTCATCTTCTCATGAGGTGAATAGCAAAATGATTTTGCTTTTTTTTTTTGCTCCAGTAAATGGGAAAATTGCTCTGGTTAGTGAGCAATTAGGGAGTTGATCTG
OFF-target sites 5:
>Os09g0426800 class=Sequence_position=chr09:15493031..15497466 (+strand)
ACCAGCCGCGTTGGGTGCAAAACCACAACCGCCAACATCTTCTAACTCCCCTCACGGCTCTCCCTCTCCCCGCACGCACAACACCGGTGAACCCCTACGTACTACTGAGCTGAGCTGAGAGGACGAAGAAGAAGATCAAGCTCGAGGGGCTTGCGATCATGGGTGCCGCATTCTTGTCGTCGTGGCCATGGGATAACCTCGGCGCGTACAAGGTATTTGTGCAGTTTGCTCCGTTCGTTGGGTTGATCGGTTTCCGGCATTGTTGATGGTGGATTGATTGTGTGTGCGCAGTATGTGTTGTACGCGCCGCTGGTGGGGAAGGCGGTGGCGGGGCGGGCGTGGGAGCGGGCGAGCCCCGACCACTGGCTGCTGCTGCTGCTCGTCCTCTTCGGCGTCAGGGCCTTGACCTACCAGCTCTGGAGCTCGTTCAGCAACATGCTCTTCGCCACCCGCCGCCGCCGCATCGTCCGCGACGGCGTCGACTTCGGCCAGATCGACAGGGAGTGGGACTGGTACGCACGAACGCGCGCTCGCCGCCGGTGCAACTTAGCTCGATCGATCAGCCAGCTGAATAATCTGTGTGTTGGGGTTTGCTTCTGCAGGGACAACTTCTTGATACTGCAGGTGCACATGGCGGCGGCGGCGTTCTACGCGTTCCCGTCGCTGCGGCACCTCCCGCTGTGGGACGCCAGGGGCCTCGCCGTCGCCGCGCTCCTCCACGTCGCCGCCACCGAGCCCCTGTTCTACGCCGCGCACAGGGCGTTCCACCGCGGCCACCTCTTCTCCTGCTACCACTTGCAACACCACTCCGCCAAGGTGCCCCAGCCATTCACAGGTAAGCATGCATCTCGCGCGCGCCGCCCACAGATCGAGTACCGATCCGCCGCCGGCGATCGATCGAAGCTAATTAACTCCGTCAATTGATCGCCGGTGCATGCGCAGCGGGGTTCGCGACGCCGCTGGAGCAGCTGGTGCTGGGGGCGCTCATGGCGGTGCCGCTGGCGGCGGCGTGCGCGGCGGGGCACGG
Fig.S2.The genomic locations and sequence information of potential off-target sites OFF-target sites 1 to 6, with the potential target sites highlighted in yellow. (Continued)

## Slide 4
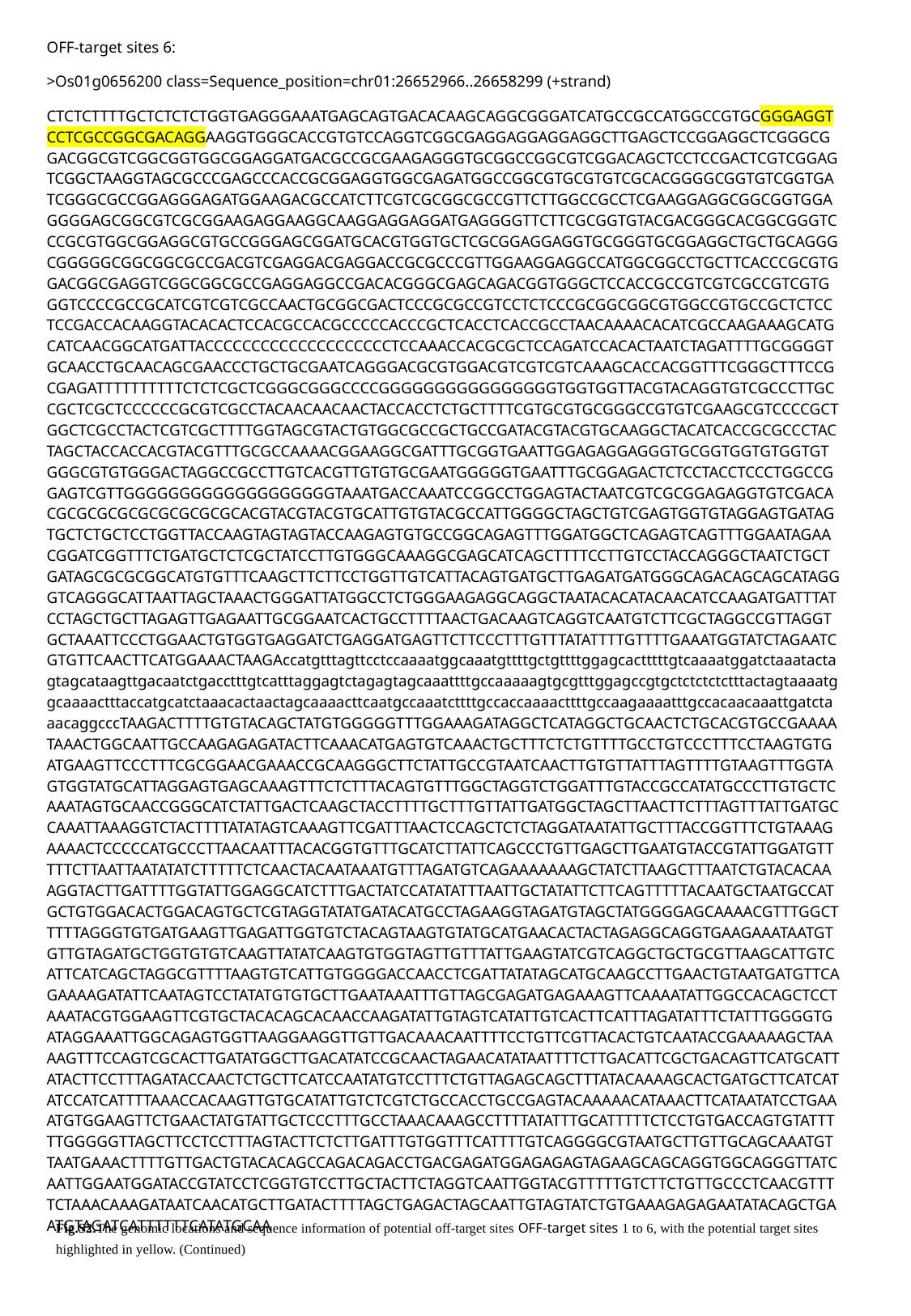

OFF-target sites 6:
>Os01g0656200 class=Sequence_position=chr01:26652966..26658299 (+strand)
CTCTCTTTTGCTCTCTCTGGTGAGGGAAATGAGCAGTGACACAAGCAGGCGGGATCATGCCGCCATGGCCGTGCGGGAGGTCCTCGCCGGCGACAGGAAGGTGGGCACCGTGTCCAGGTCGGCGAGGAGGAGGAGGCTTGAGCTCCGGAGGCTCGGGCGGACGGCGTCGGCGGTGGCGGAGGATGACGCCGCGAAGAGGGTGCGGCCGGCGTCGGACAGCTCCTCCGACTCGTCGGAGTCGGCTAAGGTAGCGCCCGAGCCCACCGCGGAGGTGGCGAGATGGCCGGCGTGCGTGTCGCACGGGGCGGTGTCGGTGATCGGGCGCCGGAGGGAGATGGAAGACGCCATCTTCGTCGCGGCGCCGTTCTTGGCCGCCTCGAAGGAGGCGGCGGTGGAGGGGAGCGGCGTCGCGGAAGAGGAAGGCAAGGAGGAGGATGAGGGGTTCTTCGCGGTGTACGACGGGCACGGCGGGTCCCGCGTGGCGGAGGCGTGCCGGGAGCGGATGCACGTGGTGCTCGCGGAGGAGGTGCGGGTGCGGAGGCTGCTGCAGGGCGGGGGCGGCGGCGCCGACGTCGAGGACGAGGACCGCGCCCGTTGGAAGGAGGCCATGGCGGCCTGCTTCACCCGCGTGGACGGCGAGGTCGGCGGCGCCGAGGAGGCCGACACGGGCGAGCAGACGGTGGGCTCCACCGCCGTCGTCGCCGTCGTGGGTCCCCGCCGCATCGTCGTCGCCAACTGCGGCGACTCCCGCGCCGTCCTCTCCCGCGGCGGCGTGGCCGTGCCGCTCTCCTCCGACCACAAGGTACACACTCCACGCCACGCCCCCACCCGCTCACCTCACCGCCTAACAAAACACATCGCCAAGAAAGCATGCATCAACGGCATGATTACCCCCCCCCCCCCCCCCCCCTCCAAACCACGCGCTCCAGATCCACACTAATCTAGATTTTGCGGGGTGCAACCTGCAACAGCGAACCCTGCTGCGAATCAGGGACGCGTGGACGTCGTCGTCAAAGCACCACGGTTTCGGGCTTTCCGCGAGATTTTTTTTTTCTCTCGCTCGGGCGGGCCCCGGGGGGGGGGGGGGGGTGGTGGTTACGTACAGGTGTCGCCCTTGCCGCTCGCTCCCCCCGCGTCGCCTACAACAACAACTACCACCTCTGCTTTTCGTGCGTGCGGGCCGTGTCGAAGCGTCCCCGCTGGCTCGCCTACTCGTCGCTTTTGGTAGCGTACTGTGGCGCCGCTGCCGATACGTACGTGCAAGGCTACATCACCGCGCCCTACTAGCTACCACCACGTACGTTTGCGCCAAAACGGAAGGCGATTTGCGGTGAATTGGAGAGGAGGGTGCGGTGGTGTGGTGTGGGCGTGTGGGACTAGGCCGCCTTGTCACGTTGTGTGCGAATGGGGGTGAATTTGCGGAGACTCTCCTACCTCCCTGGCCGGAGTCGTTGGGGGGGGGGGGGGGGGGGTAAATGACCAAATCCGGCCTGGAGTACTAATCGTCGCGGAGAGGTGTCGACACGCGCGCGCGCGCGCGCGCACGTACGTACGTGCATTGTGTACGCCATTGGGGCTAGCTGTCGAGTGGTGTAGGAGTGATAGTGCTCTGCTCCTGGTTACCAAGTAGTAGTACCAAGAGTGTGCCGGCAGAGTTTGGATGGCTCAGAGTCAGTTTGGAATAGAACGGATCGGTTTCTGATGCTCTCGCTATCCTTGTGGGCAAAGGCGAGCATCAGCTTTTCCTTGTCCTACCAGGGCTAATCTGCTGATAGCGCGCGGCATGTGTTTCAAGCTTCTTCCTGGTTGTCATTACAGTGATGCTTGAGATGATGGGCAGACAGCAGCATAGGGTCAGGGCATTAATTAGCTAAACTGGGATTATGGCCTCTGGGAAGAGGCAGGCTAATACACATACAACATCCAAGATGATTTATCCTAGCTGCTTAGAGTTGAGAATTGCGGAATCACTGCCTTTTAACTGACAAGTCAGGTCAATGTCTTCGCTAGGCCGTTAGGTGCTAAATTCCCTGGAACTGTGGTGAGGATCTGAGGATGAGTTCTTCCCTTTGTTTATATTTTGTTTTGAAATGGTATCTAGAATCGTGTTCAACTTCATGGAAACTAAGAccatgtttagttcctccaaaatggcaaatgttttgctgttttggagcactttttgtcaaaatggatctaaatactagtagcataagttgacaatctgacctttgtcatttaggagtctagagtagcaaattttgccaaaaagtgcgtttggagccgtgctctctctctttactagtaaaatggcaaaactttaccatgcatctaaacactaactagcaaaacttcaatgccaaatcttttgccaccaaaacttttgccaagaaaatttgccacaacaaattgatctaaacaggcccTAAGACTTTTGTGTACAGCTATGTGGGGGTTTGGAAAGATAGGCTCATAGGCTGCAACTCTGCACGTGCCGAAAATAAACTGGCAATTGCCAAGAGAGATACTTCAAACATGAGTGTCAAACTGCTTTCTCTGTTTTGCCTGTCCCTTTCCTAAGTGTGATGAAGTTCCCTTTCGCGGAACGAAACCGCAAGGGCTTCTATTGCCGTAATCAACTTGTGTTATTTAGTTTTGTAAGTTTGGTAGTGGTATGCATTAGGAGTGAGCAAAGTTTCTCTTTACAGTGTTTGGCTAGGTCTGGATTTGTACCGCCATATGCCCTTGTGCTCAAATAGTGCAACCGGGCATCTATTGACTCAAGCTACCTTTTGCTTTGTTATTGATGGCTAGCTTAACTTCTTTAGTTTATTGATGCCAAATTAAAGGTCTACTTTTATATAGTCAAAGTTCGATTTAACTCCAGCTCTCTAGGATAATATTGCTTTACCGGTTTCTGTAAAGAAAACTCCCCCATGCCCTTAACAATTTACACGGTGTTTGCATCTTATTCAGCCCTGTTGAGCTTGAATGTACCGTATTGGATGTTTTTCTTAATTAATATATCTTTTTCTCAACTACAATAAATGTTTAGATGTCAGAAAAAAAGCTATCTTAAGCTTTAATCTGTACACAAAGGTACTTGATTTTGGTATTGGAGGCATCTTTGACTATCCATATATTTAATTGCTATATTCTTCAGTTTTTACAATGCTAATGCCATGCTGTGGACACTGGACAGTGCTCGTAGGTATATGATACATGCCTAGAAGGTAGATGTAGCTATGGGGAGCAAAACGTTTGGCTTTTTAGGGTGTGATGAAGTTGAGATTGGTGTCTACAGTAAGTGTATGCATGAACACTACTAGAGGCAGGTGAAGAAATAATGTGTTGTAGATGCTGGTGTGTCAAGTTATATCAAGTGTGGTAGTTGTTTATTGAAGTATCGTCAGGCTGCTGCGTTAAGCATTGTCATTCATCAGCTAGGCGTTTTAAGTGTCATTGTGGGGACCAACCTCGATTATATAGCATGCAAGCCTTGAACTGTAATGATGTTCAGAAAAGATATTCAATAGTCCTATATGTGTGCTTGAATAAATTTGTTAGCGAGATGAGAAAGTTCAAAATATTGGCCACAGCTCCTAAATACGTGGAAGTTCGTGCTACACAGCACAACCAAGATATTGTAGTCATATTGTCACTTCATTTAGATATTTCTATTTGGGGTGATAGGAAATTGGCAGAGTGGTTAAGGAAGGTTGTTGACAAACAATTTTCCTGTTCGTTACACTGTCAATACCGAAAAAGCTAAAAGTTTCCAGTCGCACTTGATATGGCTTGACATATCCGCAACTAGAACATATAATTTTCTTGACATTCGCTGACAGTTCATGCATTATACTTCCTTTAGATACCAACTCTGCTTCATCCAATATGTCCTTTCTGTTAGAGCAGCTTTATACAAAAGCACTGATGCTTCATCATATCCATCATTTTAAACCACAAGTTGTGCATATTGTCTCGTCTGCCACCTGCCGAGTACAAAAACATAAACTTCATAATATCCTGAAATGTGGAAGTTCTGAACTATGTATTGCTCCCTTTGCCTAAACAAAGCCTTTTATATTTGCATTTTTCTCCTGTGACCAGTGTATTTTTGGGGGTTAGCTTCCTCCTTTAGTACTTCTCTTGATTTGTGGTTTCATTTTGTCAGGGGCGTAATGCTTGTTGCAGCAAATGTTAATGAAACTTTTGTTGACTGTACACAGCCAGACAGACCTGACGAGATGGAGAGAGTAGAAGCAGCAGGTGGCAGGGTTATCAATTGGAATGGATACCGTATCCTCGGTGTCCTTGCTACTTCTAGGTCAATTGGTACGTTTTTGTCTTCTGTTGCCCTCAACGTTTTCTAAACAAAGATAATCAACATGCTTGATACTTTTAGCTGAGACTAGCAATTGTAGTATCTGTGAAAGAGAGAATATACAGCTGAATGTAGATCATTTTTTCATATGCAA
Fig.S2.The genomic locations and sequence information of potential off-target sites OFF-target sites 1 to 6, with the potential target sites highlighted in yellow. (Continued)
