# Supplementary material for: Generation of Novel High-Quality Small-Grained Rice Germplasm by Targeting the OsVIN2 Gene
Source: Biology (Basel). 2025 Dec 30;15(1):64. doi: 10.3390/biology15010064 (PMC12784667; doi:10.3390/biology15010064)
Supplement: Supplementary file 1 [file biology-15-00064-s001.zip › Supplemental Table S1.pdf]

Supplemental information

Generation of Novel High-Quality Small-Grained Rice Germplasm by Targeting the *OsVIN2* Gene

Table S1. List of primers used in this study.

Primer	Sequence (5'-3')	Comment
sgRNA1-F	CAGTGGCGGTCCTCTCCGGCGTC	F primer for making Cas9/sgRNA construct 1
sgRNA1-R	AACGACGCCGGAGAGGACCGCC A	R primer for making Cas9/sgRNA construct 2
Cas9-F	GGGAGATCCAGCTAGAGGTC	F primer for detecting the *Cas9* gene in transgenic plants
Cas9-R	GGAAGGAGGAAGACAAGG	R primer for detecting the *Cas9* gene in transgenic plants
OsVIN2-F	CGCTTCCCTACTCCTACTCG	F primer for genotyping mutations in the OsVIN2 target sites in transgenic plants
OsVIN2-R	CATCAGCCTCACCATCTCCT	R primer for genotyping mutations in the OsVIN2 target sites in transgenic plants
Hpt-F	TACACAGCCATCGGTCCAGA	F primer for detecting the transgenic plants
Hpt-R	TAGGAGGGCGTGGATATGTC	R primer for detecting the transgenic plants
OFF1-F	CTACTCCCAGGTGGCGTACGAG ATC	F primer for PCR amplification of the potential off-target sites sgRNA1
OFF1-R	TTAGTCCACGACGTTGTTGCTG AAG	R primer for PCR amplification of the potential off-target sites sgRNA1
OFF2-F	GCAGATCGCCAACCGCAGTAAA AGC	F primer for PCR amplification of the potential off-target sites sgRNA1
OFF2-R	CCAATCCAATACCAATACAACA TAA	R primer for PCR amplification of the potential off-target sites sgRNA1
OFF3-F	GGCTCGCTGGACAGGCACCTCT TCG	F primer for PCR amplification of the potential off-target sites sgRNA1
OFF3-R	AGCATCCTCACCGCCGTCAACG GGA	R primer for PCR amplification of the potential off-target sites sgRNA1
OFF4-F	GTACGAGGAGTACGAGAAGGG GAAC	F primer for PCR amplification of the potential off-target sites sgRNA1

OFF4-R	CAACCAGATCAACTCCCTAA	R primer for PCR amplification of the potential off-target sites sgRNA1
OFF5-F	TGTTGATGGTGGATTGATTGTGTGT	F primer for PCR amplification of the potential off-target sites sgRNA1
OFF5-R	CAAGTGGTAGCAGGAGAAGAGGTGG	R primer for PCR amplification of the potential off-target sites sgRNA1
OFF6-F	GGGAAATGAGCAGTGACACAAGCA	F primer for PCR amplification of the potential off-target sites sgRNA1
OFF6-R	GCGAGCACCACGTGCATCCG	R primer for PCR amplification of the potential off-target sites sgRNA1
OFF7-F	TAACCACATCTAGCATGCAGT	F primer for PCR amplification of the potential off-target sites sgRNA1
OFF7-R	GAGCGGGGCGACGAAGACGG	R primer for PCR amplification of the potential off-target sites sgRNA1
