# Supplementary material for: Generation of Novel High-Quality Small-Grained Rice Germplasm by Targeting the OsVIN2 Gene
Source: Biology (Basel). 2025 Dec 30;15(1):64. doi: 10.3390/biology15010064 (PMC12784667; doi:10.3390/biology15010064)
Supplement: Supplementary file 1 [file biology-15-00064-s001.zip › Supplemental Table S2.pdf]

Supplemental information

Generation of Novel High-Quality Small-Grained Rice Germplasm by Targeting the *OsVIN2* Gene

Table S2. Genotypes of T_0 mutant plants in MH86

Independent T_0 transgenic plants	Mutation sequences[†]	Length of deletion/insertion (bp)[‡]	Zygosity
#1	CTCCGCCGCGGTGATCCTCGCCGTGGCGGTCCTCTCCGG- GTCAGGCTCGCCGGGCGCCCCGCGACGACGACGATGGT<br>CTCCGCCGCGGTGATCCTCGCCGTGGCGGTCCTCTCCGGCGTCAGGCTCGCCGGGCGCCCCGCGACGACGACGATGGT	-1bp/WT	Heterozygous
#2	CTCCGCCGCGGTGATCCTCGCCGTGGCGGTCCTCTCCGG- GTCAGGCTCGCCGGGCGCCCCGCGACGACGACGATGGT	-1bp	Homozygous
#3	CTCCGCCGCGGTGATCCTCGCCGTGGCGGTCCTC------ GTCAGGCTCGCCGGGCGCCCCGCGACGACGACGATGGT<br>CTCCGCCGCGGTGATCCTCGCCGTGGCGGTCCTCTCCGGCGTCAGGCTCGCCGGGCGCCCCGCGACGACGACGATGGT	-6bp/WT	Heterozygous
#4	CTCCGCCGCGGTGATCCTCGCCGTGGCGGTCCTCTCCG-- GTCAGGCTCGCCGGGCGCCCCGCGACGACGACGATGGT	-2bp	Homozygous
#5	CTCCGCCGCGGTGATCCTCGCCGTGGCGGTCCTCTC---- GTCAGGCTCGCCGGGCGCCCCGCGACGACGACGATGGT	-4bp	Homozygous
#7	CTCCGCCGCGGTGATCCTCGCCGTGGCGGTCCTCTC---- GTCAGGCTCGCCGGGCGCCCCGCGACGACGACGATGGT<br>CTCCGCCGCGGTGATCCTCGCCGTGGCGGTCCTCTCCGGCGTCAGGCTCGCCGGGCGCCCCGCGACGACGACGATGGT	-4bp/WT	Heterozygous
#9	CTCCGCCGCGGTGATCCTCGCCGTGGCGGTCCTC------ GTCAGGCTCGCCGGGCGCCCCGCGACGACGACGATGGT	-6bp	Homozygous
#11	CTCCGCCGCGGTGATCCTCGCCGTGGCGGTCCTCTCCG-- GTCAGGCTCGCCGGGCGCCCCGCGACGACGACGATGGT<br>CTCCGCCGCGGTGATCCTCGCCGTGGCGGTCCTCTCCGGCGTCAGGCTCGCCGGGCGCCCCGCGACGACGACGATGGT	-2bp/WT	Heterozygous
#12	CTCCGCCGCGGTGATCCTCGCCGTGGCGGTCCTCTCCGGCtGTCAGGCTCGCCGGGCGCCCCGCGACGACGACGATGGT<br>CTCCGCCGCGGTGATCCTCGCCGTGGCGGTCCTCTCCGG- GTCAGGCTCGCCGGGCGCCCCGCGACGACGACGATGGT	+1bp/-1bp	Bi-allelic
#13	CTCCGCCGCGGTGATCCTCGCCGTGGCGGTCCTCTCCGG- GTCAGGCTCGCCGGGCGCCCCGCGACGACGACGATGGT<br>CTCCGCCGCGGTGATCCTCGCCGTGGCGGTCCTCTCCG--GTCAGGCTCGCCGGGCGCCCCGCGACGACGACGATGGT	-1bp/-2bp	Bi-allelic
#15	CTCCGCCGCGGTGATCCTCGCCGTGGCGGTCCTCTCCGGCaGTCAGGCTCGCCGGGCGCCCCGCGACGACGACGATGGT<br>CTCCGCCGCGGTGATCCTCGCCGTGGCGGTCCTCTCCGGCGTCAGGCTCGCCGGGCGCCCCGCGACGACGACGATGGT	+1bp/WT	Heterozygous
#19	CTCCGCCGCGGTGATCC----------//---	-205bp	Homozygous
#20	CTCCGCCGCGGTGATCCTCGCCGTGGCGGTCCTCTCCGG- GTCAGGCTCGCCGGGCGCCCCGCGACGACGACGATGGT<br>CTCCGCCGCGGTGATCCTCGCCGTGGCGGTCCTCTCC--- GTCAGGCTCGCCGGGCGCCCCGCGACGACGACGATGGT	-1bp/-3bp	Bi-allelic

Table S2. Genotypes of T_0 mutant plants in MH86 (Continued)

Independent T_0 transgenic plants	Mutation sequences†	Length of deletion/insertion (bp) ‡	Zygosity
#21	CTCCGCCGCGGTGATCCTCGCCGTGGCGGTCCTCTCCGG- GTCAGGCTCGCCGGGCGCCCCGCGACGACGACGATGGT<br>CTCCGCCGCGGTGATCCTCGCCGTGGCGGTCCTCTCCGGCaGTCAGGCTCGCCGGGCGCCCCGCGACGACGACGATGGT	-1bp/+1bp	Bi-allelic
#23	CTCCGCCGCGGTGATCCTCGCCGTGGCGGTCCTCTCCGG- GTCAGGCTCGCCGGGCGCCCCGCGACGACGACGATGGT<br>CTCCGCCGCGGTGATCCTCGCCGTGGCGGTCCTCTCCGGCtGTCAGGCTCGCCGGGCGCCCCGCGACGACGACGATGGT	-1bp/+1bp	Bi-allelic
#26	CTCCGCCGCGGTGATCCTCGCCGTGGCGGTCCTCTCCGGCtGTCAGGCTCGCCGGGCGCCCCGCGACGACGACGATGGT<br>CTCCGCCGCGGTGATCCTCGCCGTGGCGGTCCTCTCCGGCaGTCAGGCTCGCCGGGCGCCCCGCGACGACGACGATGGT	+1bp/+1bp	Bi-allelic
#30	CTCCGCCGCGGTGATCCTCGCCGTGGCGGTCCTCTCCG-- -TCAGGCTCGCCGGGCGCCCCGCGACGACGACGATGGT<br>CTCCGCCGCGGTGATCCTCGCCGTGGCGGTCCTCTCCGGCGTCAGGCTCGCCGGGCGCCCCGCGACGACGACGATGGT	-3bp/WT	Heterozygous
#35	CTCCGCCGCGGTGATCCTCGCCGTGGCGGTCCTCTCCGGCtGTCAGGCTCGCCGGGCGCCCCGCGACGACGACGATGGT<br>CTCCGCCGCGGTGATCCTCGCCGTGGCGGTCCTCTCCGGCGTCAGGCTCGCCGGGCGCCCCGCGACGACGACGATGGT	+1bp/WT	Heterozygous

†Newly introduced deletions and insertions are respectively indicated by black dashes and red letters.

‡The numbers indicated the lengths of deletions or insertion compared with wild type in MH86; -: deletion; +: insertion; combined mutations are distinguished by '/'.
